# Liquid chromatograph-mass spectrometry metabolomics uncovers potential biomarkers of semen cryo-injury in goats

**DOI:** 10.5713/ab.24.0435

**Published:** 2024-10-28

**Authors:** Shun Wu, Guolin Chen, Siyuan Zhan, Linjie Wang, Jiaxue Cao, Jiazhong Guo, Li Li, Hongping Zhang, Lili Niu, Tao Zhong

**Affiliations:** 1 Farm Animal Genetic Resources Exploration and Innovation Key Laboratory of Sichuan Province, Sichuan Agricultural University, Chengdu 611130, China

**Keywords:** Cryopreservation, Goat, Liquid Chromatograph-Mass Spectrometry (LC-MS), Metabolite, Semen

## Abstract

**Objective:**

Semen cryopreservation acts a crucial role in enhancing breed improvement and conserving genetic resources. However, it often leads to decreased sperm activity and reduced pregnancy rates. Despite significant advancements in semen freezing techniques for goats, the precise factors and mechanisms causing cryo-injury remain unclear.

**Methods:**

In this study, we examined the motility characteristics of fresh semen versus frozen-thawed semen and investigated changes in the metabolite profiles of seminal plasma using liquid chromatograph-mass spectrometry.

**Results:**

A total of 364 differentially expressed metabolites (DEMs) were identified between fresh and frozen-thawed semen samples. Among these, 185 metabolites were significantly up-regulated, while 179 were down-regulated (p<0.05). The majority of these DEMs belonged to lipids and lipid-like molecules, as well as organic acids and derivatives. The Kyoto encyclopedia of genes and genomes indicated that these DEMs were primarily involved in pathways related to amino acid synthesis and metabolism. Additionally, metabolite set enrichment analysis underscored the critical role of amino acid synthesis and metabolic pathways in semen cryopreservation. Specific metabolites such as alanine, proline, phenylalanine, tryptophan, tyrosine, adenosine, citric acid, flavin adenine dinucleotide, and choline emerged as potential biomarkers for sperm cryo-injury in goats.

**Conclusion:**

These findings provide valuable insights into enhancing the quality of semen cryopreservation in goats, contributing to improved breeding and genetic resource conservation efforts.

## INTRODUCTION

In modern animal husbandry, artificial insemination (AI) and cryopreservation technologies are essential tools for enhancing herd productivity and for the long-term preservation of valuable livestock resources [1]. However, the impact of cryopreservation on sperm motility exhibits significant variability across different species. For instance, sperm from cattle [2] and pigs [3] typically maintains relatively high motility after cryopreservation and is extensively used in AI programs. In contrast, goat sperm experiences a notable decline in motility after cryopreservation, which greatly limits its use in AI [4]. Therefore, it is crucial to further investigate the underlying mechanisms of reduced motility in goat sperm following cryopreservation.

In recent years, high-throughput omics technologies have significantly advanced the study of sperm cryo-injury mechanisms [5]. Particularly, metabolomics has provided profound insights into the mechanisms of sperm cryo-injury [6]. Metabolites, as key biomarkers that reflect biological functions and physiological states, can reveal dynamic changes in cellular metabolism through their fluctuations [7]. During the cryopreservation process, changes in metabolite profile can affect sperm motility and oxidative stress levels, fluctuations in the composition and concentration of metabolites directly reflect sperm status and metabolic dynamics [8]. Seminal plasma serves as the primary carrier medium of sperm, comprising secretions from the testis, epididymis, and accessory gonads [9]. It contains a diverse array of organic and inorganic components, such as amino acids, proteins, sugars, and lipids, all of which are essential for maintaining sperm functionality [10]. There is a dynamic balance between the metabolic activities of sperm and the metabolites in the seminal plasma. These metabolites provide essential nutrients and support to the sperm [11]. Through metabolomics analysis of seminal plasma metabolites, we aim to reveal the metabolic profiles and their dynamic changes, thereby identifying biomarkers that can predict male fertility.

Currently, a wide range of metabolomic biomarkers have been identified in the seminal plasma of boars and bulls. Velho and colleagues measured the seminal plasma metabolome of 16 Holstein bulls and identified two metabolites, 2-oxoglutarate and fructose, as potential biomarkers of fertility in bulls [12]. Zhang et al, by analyzing the differences in metabolite levels in the seminal plasma of boars with varying cryopreservation qualities, found that D-aspartic acid, N-acetyl-L-glutamate, and inosine could be potential biomarkers for cryotolerance [13]. Researchers also conducted an identification analysis of seminal plasma metabolites from Duroc pigs and Liang guang Small-spotted pigs. The results showed that there are 40 different metabolites in the seminal plasma of both pig breeds, primarily focusing on energy metabolism substrates and antioxidant capacity, including fructose, carnitine, and creatinine [14]. In light of these findings, analyzing the composition and content of seminal plasma through metabolomics has become particularly important. Furthermore, reports on the use of liquid chromatograph-mass spectrometry (LC-MS) technology to study goat seminal plasma metabolomics remain relatively scarce. The heterogeneous and complex composition of seminal plasma, combined with its ability to interact with sperm, make it a valuable source of potential biomarkers [15]. Therefore, during the cryopreservation process, appropriately optimizing the composition and proportion of seminal plasma can help reduce damage to sperm caused by freezing and enhance the motility and fertility potential of sperm after thawing.

To investigate the relationship between seminal plasma and post-thaw sperm functionality, we utilized the Computer Assisted Sperm Analysis (CASA) system to assess sperm motility. Liquid chromatography coupled to tandem mass spectrometry (LC-MS/MS) technology was employed to detect metabolites in the seminal plasma of Tianfu goats before and after freezing-thawing. This study aims to identify and analyze differentially expressed metabolites (DEMs) and their associated metabolic pathways in fresh versus frozen-thawed seminal plasma, exploring their roles in the freezing and thawing process of goat sperm. This research not only provides new insights into biomarkers associated with the freezing and thawing of goat sperm but also offers potential guidance for optimizing sperm cryopreservation techniques in goats.

## MATERIALS AND METHODS

### Ethics statement

All experimental protocols involving animals were approved by the Institutional Animal Care and Use Committee of Sichuan Agricultural University, China (permit no. Dky-2022202023).

### Semen collection and dilution

Ten healthy Tianfu goats (aged 1.5 years) were selected for semen collection at the farm of Sichuan Agricultural University, Ya’an, Sichuan, China. All goats were raised under uniform management conditions. Semen was collected using the artificial vagina method and stored at 37°C. Semen was collected twice a week to ensure stable assessment of sperm quality, with each buck providing a single ejaculate. Only semen samples with sperm motility exceeding 80% and a sperm concentration more than 1 billion/mL were retained for further analyses [16]. The qualified semen samples were divided into two groups: the fresh semen control group (FP group, n = 10) and the frozen-thawed semen treatment group (CP group, n = 10). In the FP group, semen samples were initially diluted with Dilution I solution (v/v, 1:5), containing 31 g/L glucose, 46 g/L lactose, 15 g/L sodium citrate, 150 mL/L egg yolk, and 10% penicillin-streptomycin solution at 37°C. The diluted semen was then incubated at 37°C for 3 h followed by gradual cooling to 4°C. Subsequently, the semen was further diluted with Dilute II solution (94% Dilution solution I and 6% glycerol) in a 1:1 ratio.

### Cryopreservation and thawing of semen samples

The initial preparation of CP semen was similar to the FP group. The diluted semen was aliquoted into 0.25 mL straws (IMV, IMV Technologies, L’Aigle, France) and sealed with polyvinyl alcohol powder. These straws were then held 4 cm above the liquid nitrogen surface for 6–8 min before being immersed in liquid nitrogen for preservation. During thawing, the frozen semen was rapidly removed and immersed in a 37°C water bath for 30 s [17].

### Evaluation of sperm motility characteristics

Sperm motility characteristics were evaluated using the CASA system (AndroVision, v1.2.2_26; Minitube, Tiefenbach, Germany) at 250x magnification. Parameters including sperm total motility (TM, %), straight-line velocity (VSL, μm/s), curvilinear velocity (VCL, μm/s), progressive motility (PM, %), average path velocity (VAP, μm/s), straightness index (STR, %), linearity (LIN, %), and amplitude of lateral movement of sperm head (ALH, μm) were determined by analyzing at least 1,000 sperm cells across five different fields of view.

### Seminal plasma treatment and metabolite extraction

Seminal plasma samples were obtained by centrifugation at 500×g for 10 min from both groups. Subsequently, microscopic analysis (Eclipse E400; Nikon, Tokyo, Japan) was performed to ensure absence of sperm residues [13,18]. Finally, seminal plasma samples were transferred to cryotubes and stored at −80°C.

The metabolomic analysis were carried out at Shanghai Bioprofile Co., Ltd. Briefly, seminal plasma samples were thawed at 4°C. A 100 μL of plasma was transferred to a test tube, to which 400 μL of pre-cooled methanol acetonitrile (v/v, 1:1) was added. The mixture was vortexed for 10 s, followed by sonication in an ice bath for 20 min and incubation at −20°C for 2 h. Subsequently, the supernatant obtained after centrifugation (16,000×g, 20 min, 4°C) was dried using a high-speed vacuum centrifuge. For mass spectrometry analysis, a 100 μL methanol-aqueous solution (v/v, 1:1) was added to resolve the dried extract, which was then centrifuged (20,000×g, 15 min, 4°C) to obtain the supernatant. Quality control (QC) samples were prepared by combining equal amounts of all samples to ensure data quality. The preparation and analysis procedures for QC samples mirrored those used for experimental samples. The dried extracts were reconstituted in 50% acetonitrile. Each sample was filtered through a disposable 0.22 μm cellulose acetate filter, transferred to 2 mL high-performance liquid chromatography system vials, and stored at −80°C until further analysis.

### Liquid chromatography coupled to tandem mass spectrometry analysis

Samples were loaded into a 4°C automatic injector for analysis using the SHIMADZU-LC30 ultra-high-performance liquid chromatography system equipped with an ACQUITY UPLC HSS T3 column (2.1×100 mm, 1.8 μm) (Waters, Milford, MA, USA) for chromatographic separation. Both positive (+) and negative (−) ion modes of each sample were detected using electrospray ionization. Following separation by UPLC, mass spectrometry analysis was conducted using the Q Exactive Plus mass spectrometer (Thermo Scientific, Chadds Ford, PA, USA). The ionization conditions of the hybrid electrospray ionization source were set as follows: Spray Voltage: 3.8 kv (+) and 3.2 kv (−), Capillary Temperature: 320°C (±), Sheath Gas: 30 (±), Aux Gas: 5 (±), Probe Heater Temp: 350 (±), S-Lens RF Level: 50.

### Data processing

MSDIAL software was utilized for peak alignment, retention time correction, and peak area extraction from raw data. Metabolites were identified by matching their accurate mass with high precision (mass tolerance <10 ppm) and further confirmed by comparing their tandem mass spectrometry spectral matching (MS/MS) with reference spectra (mass tolerance <0.01 Da). Public databases such as the Human Metabolome Database, MassBank, and Global Natural Products Social Molecular Networking were referenced for metabolite identification. A self-constructed library of authentic metabolite standards (BP-DB) was also used. Ion peaks with more than 50% missing values within a group were excluded. Variables with less than 50% nonzero measurement values in at least one group were removed from further analysis.

Principal component analysis (PCA), partial least squares discriminant analysis (PLS-DA), and orthogonal partial least squares discriminant analysis (OPLS-DA) were employed to cluster and discriminate samples based on metabolites. Pearson’s correlation coefficient was calculated to assess the relationship between metabolites. Variable Importance for the Projection (VIP) scores were used to evaluate the influence and explanatory power of metabolite expression patterns on sample classification. Metabolites with VIP >1 were preliminarily selected.

Fold Change (FC) analysis with criteria FC >=2 or FC <=1/2, and statistical significance (p<0.05) was used to identify differential metabolites. Volcano plots were used to visually screen and highlight significant different metabolites based on Log2FC and -log10p-values. Kyoto encyclopedia of genes and genomes (KEGG) enrichment analysis of differential metabolite data was conducted using the KEGG metabolic pathway database (http://www.kegg.jp). Fisher’s exact test was applied for statistical significance, and false discovery rate correction was performed to account for multiple comparisons. Additionally, MSEA was employed to identify metabolic pathways associated with the target phenotype. Receiver operating characteristic curves were utilized to identify differential metabolites and potential biomarkers. Multiple linear regression analysis was conducted to explore correlations between sperm quality parameters and these biomarkers. A significance threshold of p<0.05 was applied for metabolic pathway analysis.

### Statistical analysis

Paired t-tests were used to assess differences in sperm motility between the FP and CP groups, with statistical significance set at p<0.05. Values were reported by mean±standard error. All multivariate data analysis and modelling were performed using R (v4.0.3) and relevant R packages.

## RESULTS

### Sperm quality parameters before and after cryopreservation

**Figure 1:**
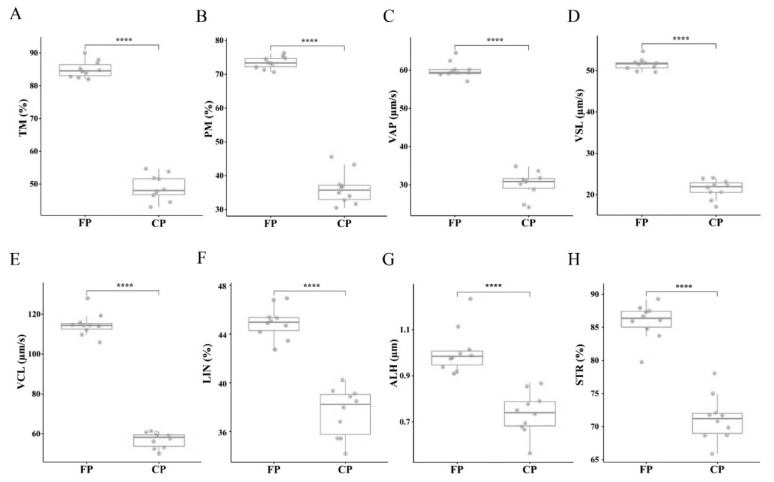
Differences in sperm motility parameters before and after cryopreservation. (A) TM, total motility. (B) PM, progressive motility. (C) VAP, average path velocity. (D) VSL, straight-line velocity. (E) VCL, curvilinear velocity. (F) LIN, linearity. (G) ALH, amplitude of lateral head displacement. (H) STR, straightness. SEM, standard error of the mean. Data presented as mean±SEM of ten replicates (n = 10). Statistical significance was determined by student’s paired t-test. * p<0.05, ** p<0.01, *** p<0.001, **** p<0.0001.

No significant differences in motility parameters were found among samples in the FP group (Figure 1; Supplementary Table S1). Following the freezing-thawing process, TM was expected to decrease, with varying degrees of decline observed among these goats. Furthermore, an assessment of motility parameters indicated that parameters such as PM, VAP, VCL, VSL, ALH, LIN, and STR were significantly higher in the FP group compared to the CP group (p<0.0001). Moreover, differences within the CP group were more pronounced than those within the FP group.

### Multivariate statistical analysis of metabolic profiles

**Figure 2:**
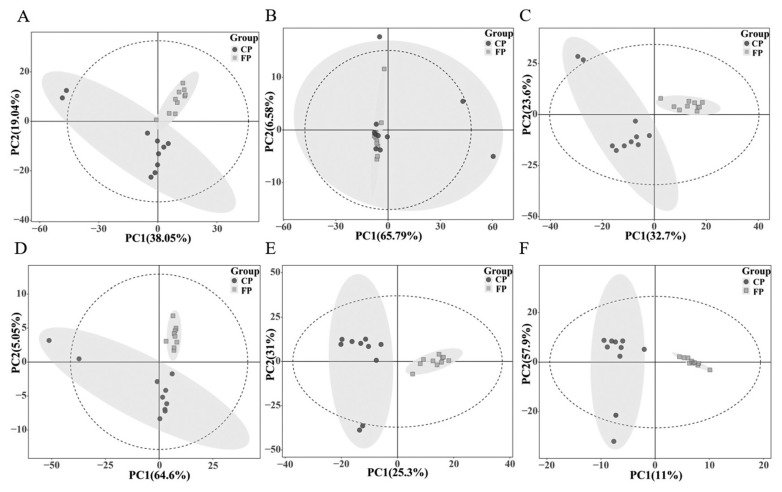
Statistical analysis of seminal plasma metabolic profiling of the FP and CP groups. (A) Principal component analysis (PCA) score plots in the positive mode. (B) PCA score plots in the negative mode. (C) Partial least squares-discriminant analysis (PLS-DA) score plot in the positive mode. (D) PLS-DA score plot in the negative mode. (E) orthogonal partial least squares discriminant analysis (OPLS-DA) score plot in the positive mode. (F) OPLS-DA score plot in the negative mode. FP, the fresh semen control; CP, the frozen-thawed semen treatment.

Unsupervised PCA was employed to visualize the overall distribution of samples, revealing distinct inter-group differences and intra-group aggregation between the FP and CP groups. While some overlap was observed between the two groups in negative ion mode, an overall trend of separation was notable. Additionally, PLS-DA and OPLS-DA analyses were conducted on both positive and negative ion modes for the FP and CP groups. Samples from the FP group predominantly clustered on the right side of the confidence interval, whereas those from the CP group were mainly positioned on the left side in both modes, indicating robust separation between the groups (Figure 2).

### Screening and identification of differentially expressed metabolites

**Figure 3:**
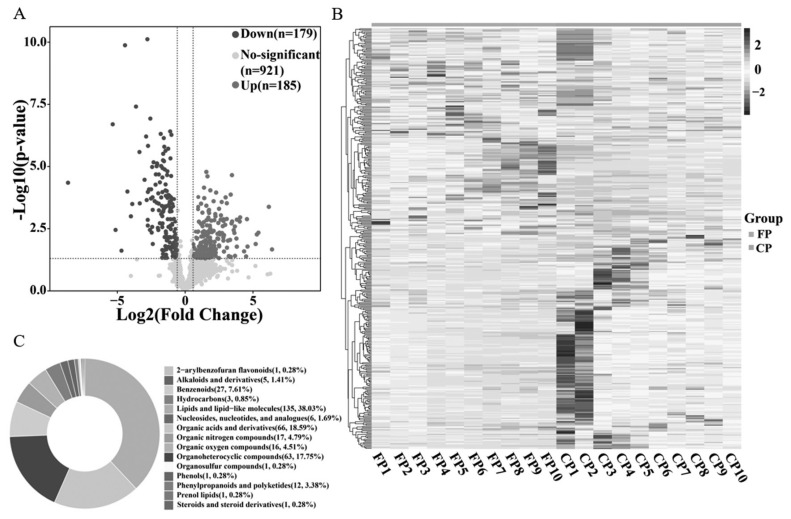
(A) Volcano map illustrating differential metabolites. (B) Heat map displaying hierarchical clustering analysis of metabolites detected in the FP and CP groups. Clustering reveals similar expression patterns. The red and blue lines denote highly and lowly expressed metabolites, respectively. (C) Classification of DEMs between the FP and CP groups. FP, the fresh semen control; CP, the frozen-thawed semen treatment; DEMs, differentially expressed metabolites.

A total of 364 DEMs were identified in seminal plasma. Among them, 185 metabolites were up-regulated, 179 metabolites were down-regulated (Figure 3A; Supplementary Table S2). To assess expression patterns of DEMs directly, hierarchical cluster analysis was performed to distinguish their patterns in seminal plasma from the FP and CP groups. The heatmap distinctly displayed clusters corresponding to samples within each group (Figure 3B). Based on the structural and functional characteristics of these metabolites, we categorized the DEMs into groups such as organic oxygen compounds, organic acids and derivatives, organoheterocyclic compounds, benzenoids, organic nitrogen compounds and organic oxygen compounds (Figure 3C).

### Analysis of metabolic pathways and identification of potential biomarkers

**Figure 4:**
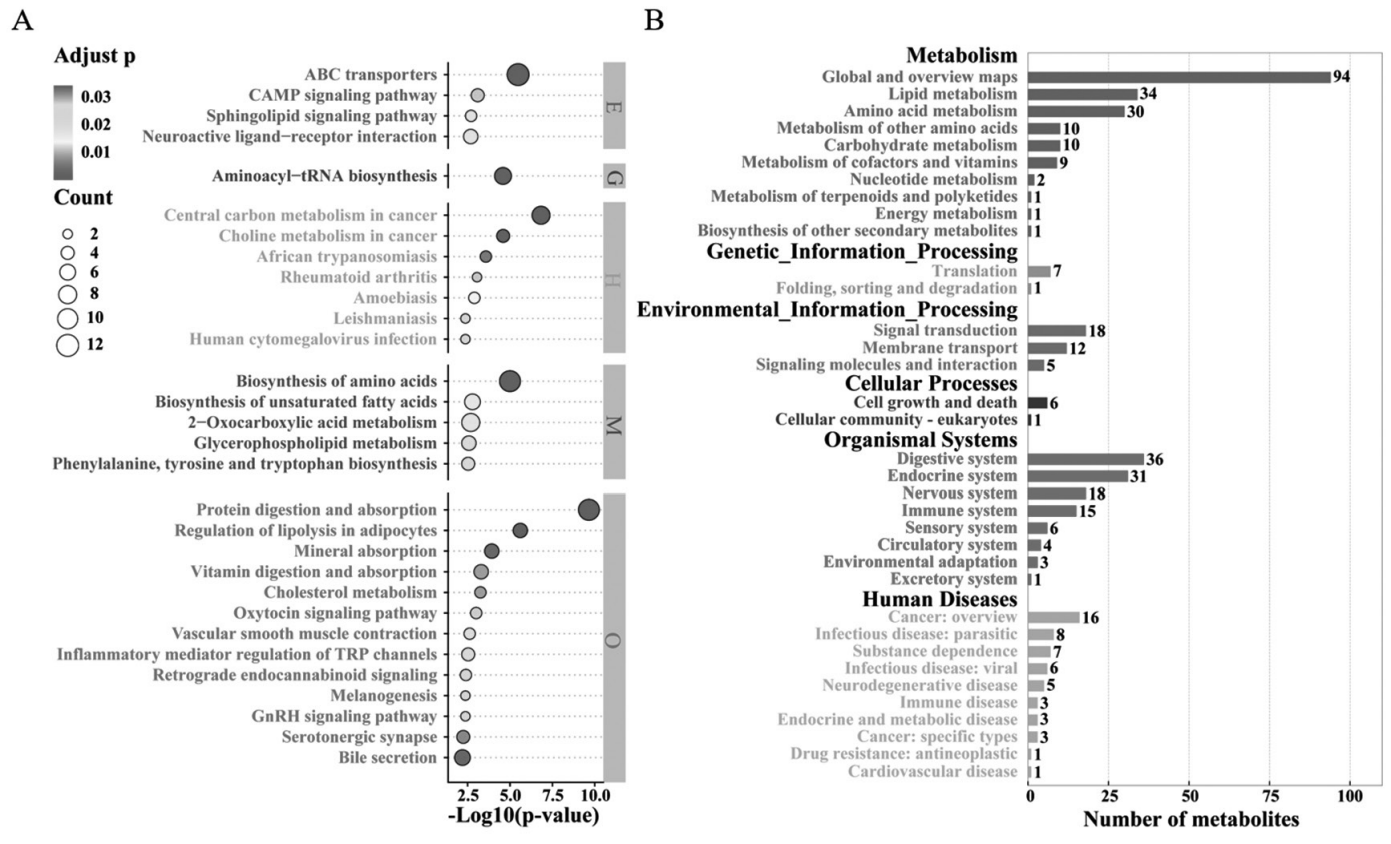
Metabolite pathway analysis of differential expression in the FP and CP groups. (A) Bubble chart showing correlations of metabolites with various metabolic pathways. (B) KEGG pathway annotation of metabolites in seminal plasma. The horizontal axis represents the number of metabolites annotated to the pathway, while the vertical axis represents the secondary classification of the KEGG pathway. FP, the fresh semen control; CP, the frozen-thawed semen treatment; KEGG, Kyoto encyclopedia of genes and genomes.

**Figure 5:**
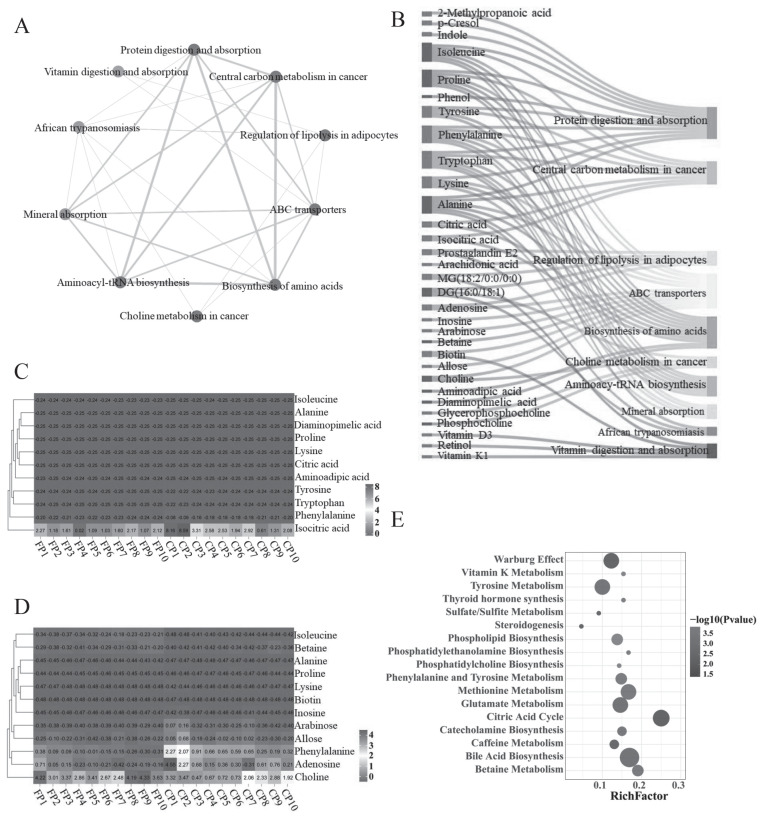
Screening of DEMs in seminal plasma. (A) Biochemical relationship network diagram depicting the top 10 significant pathways. (B) Relationship between metabolic pathways and metabolites, red denotes up-regulated metabolites, blue represents down-regulated metabolites. (C) Heatmap for the biosynthesis of amino acids pathway, where color intensity indicates correlation strength (dark red for positive, deep blue for negative). Each row and column represent different amino acids and samples, respectively. (D) ABC transporters pathway correlation heatmap. (E) Enrichment bubble plot for MSEA, each bubble represents a metabolic pathway, with size indicating to the number of metabolites and color intensity reflecting statistical significance of enrichment. FP, the fresh semen control; CP, the frozen-thawed semen treatment; DEMs, differentially expressed metabolites; MSEA, metabolite set enrichment analysis.

Pathway enrichment analysis of DEMs revealed significant enrichment in 57 metabolic pathways (p<0.05, Supplementary Table S3). These pathways include protein digestion and absorption, ABC transporters, biosynthesis of amino acids, aminoacyl-tRNA biosynthesis, and vitamin digestion and absorption (Figure 4A). A total of 364 DEMs were enriched across 35 catalogs (Figure 4B) and 161 pathways (Supplementary Table S4), spanning six categories: metabolism, genetic information processing, environmental information processing, cellular, organismal systems, and human diseases. Among the top 10 metabolic pathways, mutual regulation was observed, with various metabolites concurrently enriched within the same pathways. Conversely, this enrichment pattern was not observed in other metabolic pathways (Figures 5A, 5B). Furthermore, correlation analysis between metabolites within these pathways and samples revealed that specific metabolic pathways may regulate the same metabolites with pathway specificity. This complexity highlights the diverse relationships between metabolite content in different pathways and sample characteristics (Figures 5C, 5D).

**Figure 6:**
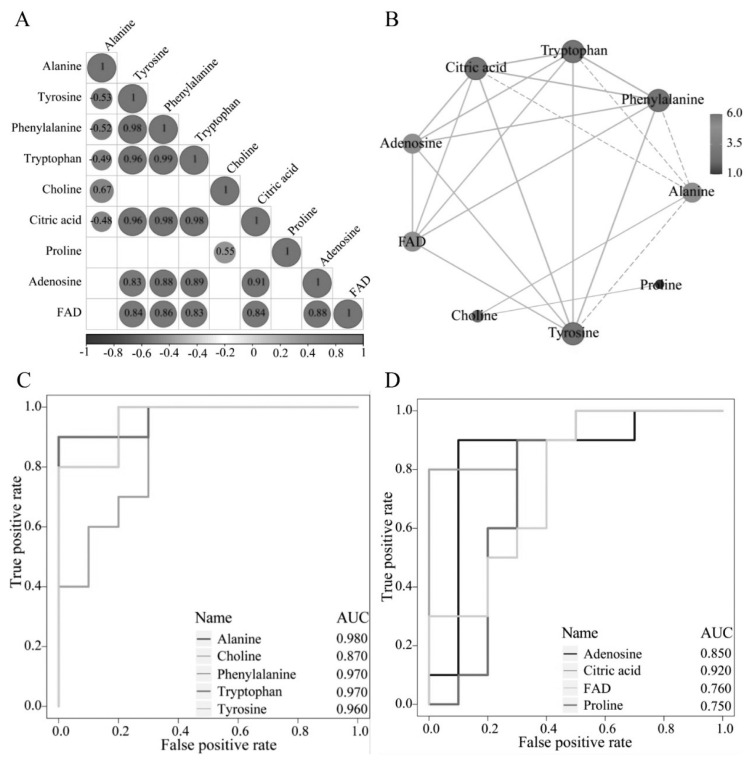
Analysis of key metabolites. (A) Correlation analysis of key metabolites, where color denotes correlation strength and direction (red for positive, blue for negative). (B) A biochemical relationship network diagram of key metabolites, where color represents the significant level (p-value) with darker red indicating smaller p-value and darker blue representing larger p-value, and circle size corresponding to fold change. (C) Receiver operating characteristic (ROC) curves for key metabolites (alanine, choline, phenylalanine, tryptophan, tyrosine), illustrating their discriminatory power. (D) ROC curves for key metabolites (adenosine, citric acid, FDA, proline), each showing the area under the curve (AUC) as a measure of discriminatory power. FAD, flavin adenine dinucleotide.

Additionally, we conducted a comprehensive enrichment analysis of all metabolites using MSEA, which revealed significant enrichment of amino acid metabolic pathways (Figure 5E). Specifically, 17 pathways showed notable enrichment (p<0.05), including phosphatidylcholine (PC) biosynthesis, vitamin K metabolism, phenylalanine and tyrosine metabolism, glutamate metabolism, and citric acid cycle (Supplementary Table S5). Importantly, we compared the results of MSEA and KEGG enrichment analyses and identified a significant overlap. Specific metabolites such as tyrosine, phenylalanine, alanine, proline, choline, tryptophan, adenosine, and citric acid were enriched in both pathways. Additionally, flavin adenine dinucleotide (FAD) was prevalent across most MSEA pathways, suggesting its crucial role in multiple aspects of cell metabolism. Subsequently, Figures 6A, 6B reveal a strong positive correlation among metabolites in seminal plasma and highlight a metabolic network centered around alanine, citric acid, and tryptophan. Moreover, compared to the FP group, the majority of differential metabolites in the CP group exhibited higher expression levels (Supplementary Figure S1). Furthermore, these nine key metabolites showed high diagnostic accuracy (AUC >0.75; Figures 6C, 6D).

## DISCUSSION

Semen cryopreservation technology plays a crucial role in preserving local goat germplasm resources and maintaining genetic diversity. Although this technique helps improve sperm survival and fertilization ability in AI, its optimization and improvement remain an ongoing area of research [19]. Seminal plasma, an essential component of semen, not only provides energy and buffering for sperm [20], but also varies in composition between species and individuals [21]. These differences in seminal plasma components likely influence the cryopreservation efficacy of sperm. However, the metabolic markers that impact the effectiveness of goat semen cryopreservation have not been clearly defined. In this study, we performed a comprehensive analysis of seminal plasma metabolites in goats using LC-MS/MS. We identified differences in seminal plasma composition between the FP and CP groups and discovered potential biomarkers that may affect semen cryopreservation. These findings offer valuable insights into exploring the molecular mechanisms underlying cryo-injury in goat sperm.

Sperm is susceptible to various stressors such as ice crystal formation and osmotic pressure during freezing [22], which significantly reduces sperm quality after thawing. In this study, goat semen was treated with a standardized cryopreservation extender and followed a consistent freezing-thawing protocol. The results showed a significant decrease in sperm motility parameters post-freezing. Furthermore, we also observed differences in cryoprotective ability among individuals within the same group, suggesting that sperm tolerance to cryopreservation may be influenced by individual variability. Given the close relationship between sperm and seminal plasma, we speculate that there may be potential interactions between sperm and seminal plasma in response to cryoprotectants. Researchers combined sperm with seminal plasma having different freezing-thawing capabilities prior to semen freezing and thawing. The findings revealed that high freezing-thawing resistant seminal plasma significantly enhanced the quality of low freezing-thawing resistant sperm after thawing [17,23]. Conversely, low freezing-thawing resistant seminal plasma decreased motility parameters and structural integrity of high freezing-thawing resistant sperm post-thawing [17,23]. Hence, the composition of seminal plasma in semen appears crucial in determining sperm’s resistance to freezing. The small molecule metabolites in seminal plasma regulate sperm physiological activities through various pathways, including sperm motility, capacitation, acrosome reaction, and survival capacity during cryopreservation [20,24–26]. Moreover, throughout the sperm cryopreservation process, their stability and functionality directly influence the survival rate and fertilization capacity of the frozen sperm [27]. In this study, we used metabolomic analysis to detect seminal plasma metabolites in the FP and CP groups. The metabolic profiles showed significant separation in both positive and negative ion modes in PCA, PLS-DA and OPLS-DA, indicating notable differences in metabolite composition between the two groups. Similar findings have been reported in the seminal plasma of pigs and donkeys [13,28], suggesting that sperm cryopreservation has a substantial impact on seminal plasma metabolites.

A total of 364 DEMs were identified between the FP and CP groups, including 185 up-regulated metabolites and 179 down-regulated metabolites. Metabolites are typically classified into amino acids, carbohydrates, lipids, nucleotides, vitamins, and minerals. The presence of these DEMs suggests that cryopreservation significantly impacts the metabolic environment of seminal plasma, potentially reflecting the metabolic pathway remodeling that sperm undergo during freezing to adapt to environmental changes. In this study, we found that lipids and lipid-like molecules accounted for the largest proportion in seminal plasma, followed by organic acids and derivatives, consistent with previous observations in pig seminal plasma [29]. Lipids and lipid-like molecules play crucial roles in regulating sperm survival, motility, and fertilization ability [30,31]. They directly influence the stability and integrity of the sperm membrane [32], serve as an energy source for sperm [33], and contribute significantly to cell signaling processes [34]. To explore the impact of DEMs on sperm cryopreservation, we first conducted KEGG enrichment analysis highlighted tryptophan, proline, alanine, isoleucine, phenylalanine, choline, adenosine, and other amino acids as potential biomarkers for cryo-injury in goat sperm. To enrich the outcomes of KEGG enrichment analysis, we conducted MSEA by incorporating all identified metabolites from our metabolomic dataset. This approach aimed to explore metabolites exhibiting subtle changes in abundance yet holding vital regulatory significance for the organism. The results of MSEA revealed significant enrichment of tryptophan, proline, alanine, phenylalanine, choline, adenosine, and citric acid. Furthermore, FAD was notably enriched within the MSEA pathway. Consequently, comprehensive enrichment analysis of all metabolites has become increasingly imperative. Importantly, we observed close associations among nine metabolites enriched by both enrichment methods.

Various amino acids identified in seminal plasma serve multiple biological functions, such as mitigating free radical formation and shielding cells from degradation [35]. In this study, tryptophan, as an essential amino acid, may improve sperm quality and reproductive capacity through multiple pathways. A study by A. Sh. Ahmad showed that the addition of 0.150 g of tryptophan improved semen quality in Iraqi Shami bucks [36]. Proline, a multifunctional amino acid, plays a crucial role in carbon and nitrogen metabolism, osmotic regulation, oxidative stress protection, and cell signaling [37]. Studies have shown that proline can significantly improve the quality of thawed ram sperm [38] and donkey sperm [39]. Phenylalanine, as one of the essential amino acids for the human body, is mostly oxidized into tyrosine by the catalytic action of phenylalanine hydroxylase [40]. Together with tyrosine, it synthesizes important neurotransmitters and hormones, playing a role in glucose metabolism and fat metabolism. Lahnsteiner’s study found that 2.5 mmol/L of phenylalanine has a positive effect on sperm motility [41]. Kundu’s study demonstrated that glutamine, proline, glycine, and alanine at concentrations of 100 to 150 mmol/L exhibit significant cryoprotective potential, with 135 mmol/L of alanine showing the best cryoprotective effect [42]. In addition to these amino acids that have been proven to enhance and sustain sperm motility, choline, as a major component of cell membrane, especially PC, plays a vital role in preserving the structural integrity and function of cells [43]. Researches have highlighted the essential role of PC in sperm motility and fertilization [44]. Furthermore, citric acid, as a key substrate in the mitochondrial tricarboxylic acid cycle, provides an efficient way of sperm to generating energy, supporting sperm metabolism [45]. At the same time, FAD, an essential cofactor in cellular metabolism, participates in mitochondrial process and promotes intracellular energy production, with its deficiency significantly impairing sperm function [46]. Adenosine, a purine nucleoside formed by the glycosidic bond between the purine base adenine and D-ribose [47,48], has been shown in studies by Masino et al to act as a potential antioxidant, released into different tissues under oxidative stress, protecting the sperm plasma membrane from oxidative damage [49]. Furthermore, we analyzed the expression levels of 9 metabolites to evaluate their potential as biomarkers. Based on the significance analysis of p-value, proline (p = 0.0487), tryptophan (p = 0.0014), alanine (p = 0.0005), phenylalanine (p = 0.0011), choline (p = 0.0017), adenosine (p = 0.0412), citric acid (p = 0.0046), and tyrosine (p = 0.0014) all showed significant expression differences. Although FAD (p = 0.0583) showed somewhat inadequate statistical significance, it still warrants further investigation. Importantly, in the CP group, the metabolic pathways of these metabolites exhibit significant changes, particularly in protein metabolism, energy supply, signal transduction, and oxidative stress response. For instance, amino acids such as tryptophan, proline, tyrosine, alanine, and phenylalanine are fundamental to sperm protein metabolism in semen. However, they also play unique roles in specific metabolic pathways. For example, adenosine regulates energy metabolism through the cAMP signaling pathway and participates in processes such as lipolysis. FAD is involved in oxidative phosphorylation and energy metabolism. Therefore, understanding the roles of these metabolites and their changes during the freezing process not only helps improve semen freezing and preservation techniques but also provides new biomarkers for future research to enhance the quality and reproductive effectiveness of frozen semen.

## CONCLUSION

In this study, we utilized LC-MS/MS to characterize the metabolomic profiles of goat seminal plasma and discerned differences in metabolites between the FP and CP groups. Seminal plasma predominantly featured lipid and lipid-like molecules, as well as organic acids and derivatives. The observed decline in sperm motility within the CP group could likely be attributed to an imbalance in amino acid synthesis and metabolism. Furthermore, metabolic pathways involving citric acid, FAD, and choline were identified as potential influencers of sperm energy absorption. Nine potential biomarkers were identified including alanine, proline, phenylalanine, tryptophan, tyrosine, adenosine, citric acid, FAD, and choline, that may associate with cryodamage in goat sperm and potentially modulate sperm motility during semen cryopreservation.

## Data Availability

Upon reasonable request, the datasets of this study can be available from the corresponding author.

## SUPPLEMENTARY MATERIAL

**Supplementary Table S1.** Comparison of sperm motility parameters before and after cryopreservation

**Supplementary Table S2.** List of identified differential expressed metabolites (DEMs)

**Supplementary Table S3.** Significant KEGG metabolic pathways based on p<0.05

**Supplementary Table S4.** Enriched KEGG metabolic pathways and their classifications

**Supplementary Table S5.** Significantly enriched metabolic pathways from MSEA

**Supplementary Figure S1.** Relative abundance of nine selected metabolites in the FP and CP groups
