# Supplementary material for: Liquid chromatograph-mass spectrometry metabolomics uncovers potential biomarkers of semen cryo-injury in goats
Source: Anim Biosci. 2024 Oct 28;38(4):629–40. doi: 10.5713/ab.24.0435 (PMC11917422; doi:10.5713/ab.24.0435)
Supplement: Supplementary file 1 [file ab-24-0435-Supplementary-Table-1.pdf]

Supplementary materials

Supplementary Table S1. Comparison of sperm motility parameters before and after cryopreservation.

Groups	TM (%)	PM (%)	VAP (μm/s)	VSL (μm/s)	VCL (μm/s)	LIN (%)	ALH (μm)	STR (%)
FP	85.02 ± 0.82^{A}	73.42 ± 0.56^{A}	60.01 ± 0.65^{A}	51.50 ± 0.46^{A}	114.80 ± 1.85^{A}	44.93 ± 0.42^{A}	1.01 ± 0.03^{A}	85.87 ± 0.85^{A}
CP	48.87 ± 1.22^{B}	36.33 ± 1.53^{B}	30.12 ± 1.09^{B}	21.41 ± 0.71^{B}	56.88 ± 1.22^{B}	37.69 ± 0.66^{B}	0.74 ± 0.03^{B}	71.22 ± 1.08^{B}

Data presented as mean ± SEM (Standard error of mean). Different superscript lowercase letters indicate significant differences ($p<0.05$), whereas different superscript uppercase letters indicates extremely significant differences ($p<0.001$), the same superscript lowercase letter indicates that the difference is not significant ($p>0.05$).
