# Supplementary material for: Liquid chromatograph-mass spectrometry metabolomics uncovers potential biomarkers of semen cryo-injury in goats
Source: Anim Biosci. 2024 Oct 28;38(4):629–40. doi: 10.5713/ab.24.0435 (PMC11917422; doi:10.5713/ab.24.0435)
Supplement: Supplementary file 2 [file ab-24-0435-Supplementary-Table-2.pdf]

Supplementary Table S2. List of identified differential expressed metabolites (DEMs).

Metabolite name	pvalue	FC	Log2FC	Significant
N-(benzimidazol-2-ylmethyl)-2-(4-hydroxyquinazolin-2-ylthio)acetamide	7.66106E-11	0.144884895	-2.7870209	DOWN
PC(16:0/18:1)	1.33732E-10	0.046221875	-4.435280406	DOWN
1H-Indene-1,2(3H)-dione	3.87686E-07	0.46434545	-1.106729597	DOWN
1-Dodecyl-2-pyrrolidinone	1.18522E-07	0.168482428	-2.56932996	DOWN
PC(18:0/20:4)	2.00758E-07	0.024646055	-5.342499423	DOWN
Spiroxamine	3.90011E-08	0.080887108	-3.627946403	DOWN
4-Hydroxybenzaldehyde	5.31994E-07	0.494642224	-1.015542698	DOWN
3,4-Diaminopyridine	7.19278E-07	0.334905009	-1.578176141	DOWN
-N-(3-methoxybenzyl)octadeca-9,12-dienamide	4.89108E-07	0.286235203	-1.804726981	DOWN
Pyridine	6.22371E-07	0.135824322	-2.880186245	DOWN
Mls001076857	1.9276E-06	0.420323724	-1.250427208	DOWN
-N-(2-hydroxyethyl)hexadec-9-enamide	1.60427E-06	0.343376964	-1.542134836	DOWN
Methyl sterculate	2.34493E-06	0.36542861	-1.452338507	DOWN
4-[(2Z)-3-(2,4-dichlorophenyl)prop-2-enoyl]phenyl 4-methylbenzoate	8.12706E-06	0.239395144	-2.06253421	DOWN
Desethylspiroxamine	1.48027E-06	0.149705322	-2.739802586	DOWN
Cloperastine hydrochloride	5.89804E-06	0.333362049	-1.584838223	DOWN
3,4-Dihydropyridine	2.61418E-06	0.096684924	-3.370565238	DOWN
LPE(16:0)	3.07505E-06	0.325216026	-1.620529744	DOWN
2-(2,9-dimethyl-6-oxohydropurin-8-ylthio)-N-(3-fluoro-4-methylphenyl)acetamide	6.7609E-06	0.198666008	-2.331583048	DOWN
P-Menthatriene	9.75615E-06	0.232521272	-2.104565391	DOWN
MG(18:2/0:0/0:0)	1.64963E-05	2.949549408	1.560494576	UP
4,4'-Dipyridyl disulfide	4.71212E-06	0.471288153	-1.085318678	DOWN
N-butyl-N-(1-(cyclohexylamino)-1-oxobutan-2-yl)-4-methylpentanamide	2.74683E-05	0.31241664	-1.678456796	DOWN
Isoleucine	7.48211E-06	0.237281961	-2.075325669	DOWN
Palmitoyl glycerol	6.13178E-06	0.338463745	-1.562926791	DOWN
N-Acetylproline	5.62317E-06	0.322430224	-1.632941108	DOWN
Phenol	9.591E-06	0.292902639	-1.771506905	DOWN
Pseudotropine	1.31237E-05	0.351562981	-1.508144929	DOWN
4-Vinylcyclohexene	1.16107E-05	0.315755527	-1.663120108	DOWN
Dodemorph	1.04341E-05	0.18484859	-2.435584056	DOWN

Alanine betaine	2.07999E-05	0.260251878	-1.942019523	DOWN
Cyclopentylamine	2.22049E-05	10.91527838	3.448277019	UP
(6E,9E,12E)-Hexadeca-6,9,12-trienoylcarnitine	2.07463E-05	0.223587751	-2.161086943	DOWN
3,7-Dimethyl-1-propargylxanthine	0.000119907	0.253044011	-1.982539768	DOWN
Naftopidil	0.000134648	10.23840931	3.355919684	UP
N-[4-[(9S,13R)-16-hydroxy-7,9,13-trimethyl-5-oxapentacyclo[10.8.0.02,9.04,8.013,18]icosa-6,18-dien-6-yl]-2-methylbutyl]acetamide	3.41721E-05	0.125436449	-2.99497147	DOWN
1-Methyl-4-isobutylbenzene	5.36613E-05	0.220195702	-2.183141785	DOWN
Monoolein	4.33123E-05	0.274487463	-1.865187839	DOWN
N-(10-hydroxy-10-methylundecyl)acetamide	0.000101528	0.052224342	-4.259133766	DOWN
(E)-N-butyl-N-(1-(cyclohexylamino)-1-oxobutan-2-yl)but-2-enamide	1.55858E-05	0.402596469	-1.312593577	DOWN
LPC(20:3)	2.2032E-05	0.397374932	-1.33142723	DOWN
p-Cresol	2.48678E-05	3.125081906	1.643894002	UP
2,4-Dichlorobenzoic acid	0.000194443	0.174284553	-2.52048339	DOWN
Isostearic acid	0.000174143	7.892004903	2.980391852	UP
5-Deoxykievitone	0.000111595	4.855787034	2.279705149	UP
DG(16:0/18:1)	4.49448E-05	0.002510551	-8.637780499	DOWN
Creatine riboside	0.000151266	0.371443941	-1.428783603	DOWN
malyngamide I	8.25646E-05	0.175502278	-2.510438337	DOWN
LPE(18:0)	5.24916E-05	0.367236905	-1.445217047	DOWN
2-Acetylpyrrolidine	8.29859E-05	0.157597572	-2.665682787	DOWN
3, 5-Tetradecadiencarnitine	0.000139807	0.426526958	-1.229291168	DOWN
4-({4-[(3-chlorophenyl)methyl]piperazinyl}methyl)-7-methylchromen-2-one	0.000154817	0.453298026	-1.141468216	DOWN
Monopalmitolein	0.000186563	2.649373237	1.405651101	UP
Phosphatidyl serine	0.000310258	0.258710743	-1.950588129	DOWN
Icos-19-Ene-1,2,4-Triol	0.000174574	0.484634146	-1.045032037	DOWN
6,10,14-Trimethyl-5,9,13-pentadecatrien-2-one	0.000168375	1.952508512	0.965328838	UP
Ethyl chrysanthemate	0.000141578	0.424349521	-1.236675045	DOWN
Indole	7.08273E-05	2.618474859	1.388726753	UP
1,3,5-Hexatriene	0.000203667	0.569513641	-0.812197697	DOWN
Methyl Deoxycholate	0.000248511	4.267336823	2.093335988	UP
Terpestacin	0.000105212	0.148762081	-2.748921259	DOWN
N-Nitrosopiperidine	9.12722E-05	0.365188005	-1.453288717	DOWN

N-Dimethylaminoprostaglandin F2alpha	0.000299763	8.393558398	3.069282562	UP
Tenuazonic acid	0.000107044	0.312312987	-1.67893553	DOWN
Dimethyl sulfoxide	0.000376224	2.952520455	1.561947055	UP
Indole-3-carboxyaldehyde	0.000181703	0.404636236	-1.305302571	DOWN
Ethoxycarbonylmethyl ethyl phthalate	0.000448324	0.466159095	-1.101105678	DOWN
Peimisine	0.000172841	0.18888624	-2.404410489	DOWN
LPE(17:0)	0.000311891	2.252962181	1.171823096	UP
epsilon-Decalactone	0.000186195	0.497217132	-1.00805209	DOWN
DL-Cycloserine	0.000258865	0.454521539	-1.137579433	DOWN
Xanthopterin	0.000457109	0.492350495	-1.022242386	DOWN
p-Aminophenetole	0.000398224	0.543397356	-0.87992055	DOWN
Benthiavalicarb isopropyl	0.000223002	0.139915133	-2.837376089	DOWN
Glycocholate	0.000302354	0.098161146	-3.348704095	DOWN
Oleic acid	0.000415931	1.940341817	0.956310825	UP
1-Phenylethanol	0.000266831	6.459811402	2.691492045	UP
Myristoleic acid	0.000240501	0.482568606	-1.051194033	DOWN
(3E,5Z)-1,3,5-Heptatriene	0.000403686	0.351586789	-1.508047235	DOWN
Photopyrone G	0.000523434	1.929467576	0.9482028	UP
Chenodeoxycholylhistidine	0.000320901	0.066972316	-3.900291334	DOWN
Boc-glycine	0.000554874	3.380240364	1.757125838	UP
Eicosadienoic acid	0.001622041	11.00552081	3.460155513	UP
Alanine	0.000508296	0.335035942	-1.577612223	DOWN
Furanone A	0.000427677	0.359740518	-1.474971433	DOWN
Tromethamine	0.000500493	5.641796884	2.496154727	UP
Prostaglandin E2	0.001747041	2.57268002	1.363272031	UP
8-Hydroxy-9,10-epoxystearic acid	0.000320086	0.527400558	-0.923028997	DOWN
brachystamide	0.000576046	0.261489853	-1.93517313	DOWN
5beta-Cholanic Acid	0.001010149	4.696351533	2.231540402	UP
Chenodeoxycholylalanine	0.000383683	0.305104796	-1.712623239	DOWN
Coronaric acid	0.000918992	6.218914372	2.636662753	UP
9-Methoxyellipticine	0.000820411	0.293285883	-1.769620465	DOWN
methyl (Z)-pentadec-10-enoate	0.000545858	0.467201928	-1.097881866	DOWN

O-Arachidonoylglycidol	0.000553379	0.190128173	-2.394955772	DOWN
Sulochrin	0.00067735	0.317205946	-1.656508278	DOWN
Vitamin K1	0.00041729	71.85486428	6.167013919	UP
4-Oxogazaniaxanthin	0.001111006	16.58016464	4.051386428	UP
Taurodeoxycholic acid	0.000715605	2.410721997	1.26946529	UP
1,7-Dimethyl-7-(4-methyl-3-penten-1-yl)bicyclo[2.2.1]heptan-2-ol	0.000408582	0.369957488	-1.434568596	DOWN
Oleoylcarnitine	0.000628699	0.326870612	-1.613208421	DOWN
Trolamine	0.001254294	0.567536494	-0.81721493	DOWN
N-Oleoyl Phenylalanine	0.000616183	3.309111546	1.726443924	UP
PC(17:0/16:1)-D5	0.001899195	0.27462909	-1.864443646	DOWN
gamma-Glutamylphenylalanine	0.000739294	3.400518578	1.765754774	UP
PC(O-16:0/18:1)	0.0035699	0.253466255	-1.980134404	DOWN
Moracin P	0.001541601	7.081640256	2.824083557	UP
Benzofuran	0.000392651	0.383223764	-1.383741069	DOWN
Batilol	0.002516704	2.424498021	1.277686076	UP
Diatoxanthin	0.000636352	0.503726313	-0.989288001	DOWN
Ricinoleic acid	0.001934735	10.68318384	3.417269764	UP
Isolongifolol	0.001316003	0.520558488	-0.941867826	DOWN
Phosphocholine	0.001005576	0.062441579	-4.001349172	DOWN
Ethyl oleate	0.00131383	0.469513676	-1.090760915	DOWN
Tyrosine	0.001435043	2.978668778	1.574667708	UP
Majusculamide A/B	0.001396507	0.147496726	-2.761245161	DOWN
Vitamin D3	0.000815796	4.657017094	2.219406178	UP
Phenylalanine	0.00113238	3.266116476	1.707576241	UP
8-[(2-hydroxyethyl)methylamino]-1,3,9-trimethyl-1,3-dihydropurine-2,6-dione	0.001939502	0.314611707	-1.66835574	DOWN
LPC(17:1)	0.001997603	2.234701072	1.16008186	UP
gamma-linolenyl carnitine	0.000990715	0.31053794	-1.687158555	DOWN
Vitispirane	0.001305853	23.48646466	4.55375766	UP
N-Fructosyl isoleucine	0.00128123	8.361444664	3.063752228	UP
4-{[5-(7-Hydroxy-5,5,8a-trimethyl-2-methylenedecahydro-1-naphthalenyl)-3-methylpentyl]oxy}-4-oxobutanoic acid	0.001368476	14.01389406	3.80878599	UP
(2E,4E)-Octadeca-2,4-dienoylcarnitine	0.001307477	0.411132761	-1.282323756	DOWN
Lysine	0.001369344	6.131155977	2.616159107	UP

Tryptophan	0.001352236	3.199661491	1.677919283	UP
Phytosphingosine	0.002688939	0.425944839	-1.231261485	DOWN
smenospongiarine	0.001627994	16.23202009	4.02077065	UP
3-(Tetradecanoylamino)propyl(carboxymethyl)dimethylammonium	0.002375084	3.525232643	1.817718469	UP
PC(16:0/22:6)	0.002368855	0.148088787	-2.755465685	DOWN
Protoporphyrin IX	0.001852048	5.446720367	2.445387802	UP
Bilobalide	0.001441258	4.431308991	2.147732928	UP
Monogynol	0.003295628	0.481724869	-1.053718691	DOWN
5,6-Dihydroxylutein	0.002579246	12.64026224	3.659954489	UP
Choline	0.001648354	0.600187364	-0.736515149	DOWN
Adiphenine Hydrochloride	0.002317946	3.032090136	1.600312642	UP
12-[(Cyclohexylcarbamoyl)amino]dodecanoic acid	0.001979569	0.515636294	-0.95557428	DOWN
Dibutyl phthalate	0.002205758	7.717039043	2.948047405	UP
2-(14-Methylpentadecanoylamino)-3-phenylpropanoic acid	0.002499446	6.824918608	2.770811841	UP
2-(4-Ethenyl-2,5-Dimethylhexa-2,5-Dienoxy)-6-(Hydroxymethyl)Oxane-3,4,5-Triol	0.006562357	0.563930837	-0.82640986	DOWN
Flunixin	0.001500283	3.807884971	1.928989898	UP
4-Methylproline	0.002231518	0.521552393	-0.939115907	DOWN
Tetradec-5-ynoic acid	0.003537543	0.165442371	-2.59559933	DOWN
Bicyclogermacrene	0.004334885	3.924875602	1.972646929	UP
7-Ketocholesterol	0.004766737	5.61975267	2.490506638	UP
3-Hexadecyl-4-methoxy-5-methyloxolan-2-one	0.004107223	3.217320826	1.685859806	UP
(2R,4aR,7S,8S,8aR)-8-[(Z)-5-hydroxy-3-methylpent-3-enyl]-4,4,7,8a-tetramethyl-2,3,4a,5,6,8-hexahydro-1H-naphthalene-2,7-diol	0.003237444	0.603468748	-0.728649035	DOWN
Lupinine	0.003618526	11.93046646	3.576578546	UP
beta-Amyrin	0.003871732	4.282177069	2.098344453	UP
Isoapetalic acid	0.005379872	5.923115382	2.56635619	UP
Korseveriline	0.006045952	2.626643929	1.39322064	UP
Mmv688796	0.005077183	5.220340244	2.38414384	UP
Theasaponin F1	0.00406845	0.371879986	-1.427090987	DOWN
2-[[(2R)-2-dodeca-4,7,10-trienoyloxy-3-hexadecoxypropoxy]-hydroxyphosphoryl]oxyethyl-trimethylazanium	0.009371373	0.390968318	-1.35487639	DOWN
Incensole acetate	0.005743404	7.262045444	2.860375958	UP
Lauryldiethanolamine	0.002383144	0.469506959	-1.090781553	DOWN

2-(N-butylpropionamido)-N-cyclohexylbutanamide	0.004123684	0.573155993	-0.803000251	DOWN
h_55_metenolone-m	0.005370643	38.96683667	5.284174913	UP
2H-benzo[3,4-d]1,3-dioxolen-5-yl 4-[(3,5-dimethyl-1-phenylpyrazol-4-yl)methyl] piperazinyl ketone	0.004372529	2.436335881	1.284713041	UP
Isonerylgeraniol-18-oic acid	0.004567395	42.15251772	5.397546899	UP
Palmitoylcarnitine	0.002737968	0.310359919	-1.687985839	DOWN
Lithocholic acid	0.004541995	5.773674805	2.529489852	UP
PFSM-perfluoroalkyl_sulfonamide_Et	0.005093857	0.603157105	-0.729394263	DOWN
Platelet-activating factor	0.007302152	0.579773442	-0.786438846	DOWN
Pyroglutamic acid	0.004686684	12.36916682	3.628676419	UP
Citric acid	0.00464556	3.32681773	1.734142826	UP
Trans-Hexadec-2-Enoyl Carnitine	0.002931728	0.247382663	-2.0151837	DOWN
LPC(P18:1)	0.002079567	0.30660492	-1.705547247	DOWN
3-Methyl-4-phenyl-3-buten-2-one	0.005031522	5.882927104	2.55653416	UP
Desmosterol	0.006522813	2.284940701	1.192156725	UP
Octadeca-9,12-dienal	0.00810876	1.828216341	0.870436801	UP
3-Hydroxy-11Z-octadecenoylcarnitine	0.004808387	0.375994347	-1.411217123	DOWN
Glutamylleucine	0.005053752	2.573847972	1.363926841	UP
Tetradecyldiethanolamine	0.006765745	0.403224443	-1.310345	DOWN
Cholestane	0.00710439	0.482598605	-1.05110435	DOWN
Triethyl Citrate	0.007055919	7.03475316	2.814499802	UP
8-[(cyclohexylethylamino)methyl]-7-hydroxy-4-phenylchromen-2-one	0.009001916	3.555021368	1.829858231	UP
Taurocholic acid	0.00477528	2.712636419	1.439695693	UP
Vincosamide	0.004723821	6.590888782	2.720473026	UP
2,11,16-trihydroxy-9-(hydroxymethyl)-5,5-dimethyl-14-methylidenetetracyclo[11.2.1.0,?.0?,?]hexadecan-15-one	0.011752486	2.218671892	1.149696331	UP
Gly-Trp-Gly	0.004305995	1.906051803	0.93058733	UP
3,4,5-Trimethoxyhydrocinnamic acid	0.01485831	0.643443327	-0.63611501	DOWN
Glycocyamine	0.010596126	4.501183316	2.170304321	UP
Dodecatetraenoic acid isobutylamide, (2E,4E)-	0.005778588	0.591087415	-0.758556591	DOWN
1,2-Benzoquinone	0.005378051	2.968668874	1.569816184	UP
8-{5-[(3E)-2-(acetyloxy)pent-3-en-1-yl]-1-hydroxy-4-oxocyclopent-2-en-1-yl}octanoic acid	0.01008423	3.709325431	1.891156846	UP

Cyclopenta[c]pyran-4,7-dicarboxylic acid, 1-(beta-D-glucopyranosyloxy)-1,4a,5,6,7,7a-hexahydro-5,6-dihydroxy-, dimethyl ester, (1S,4aS,6R,7R,7aS)-	0.007204781	3.559721433	1.831764347	UP
Prolylproline	0.007297058	0.5833582	-0.777546079	DOWN
Dehydrophytosphingosine	0.006855191	0.349664536	-1.515956613	DOWN
(8aR,12S,12aR)-12-hydroxy-4-methyl-4,5,6,7,8,8a,12,12a-octahydro-1H-3-benzoxecine-2,9-dione	0.012987949	0.190874264	-2.389305503	DOWN
1-O-Hexadecyl-sn-glycero-3-phosphocholine	0.009169464	0.678487783	-0.559605257	DOWN
Diaminopimelic acid	0.007418897	0.430078636	-1.217327627	DOWN
1,2,3,4,Tetrahydro-1,5,7-trimethylnapthalene	0.00917886	2.855194289	1.513588921	UP
[4-(6,7-dimethoxy(1,2,3,4-tetrahydroisoquinolyl))phenyl]dimethylamine	0.009200712	0.620222153	-0.689143037	DOWN
Glutamyltryptophan	0.016788567	0.499843457	-1.00045176	DOWN
LPC(18:2)	0.006411772	0.565562398	-0.82224189	DOWN
(1R,2R,4aS,8aS)-1-((S)-3-hydroxy-3-methylpentyl)-2,5,5,8a-tetramethyldecahydronaphthalen-2-ol	0.007075368	0.444271021	-1.170488056	DOWN
Arachidonic acid	0.007148739	1.74895419	0.806492502	UP
8,15-Eperuanediol	0.014577981	2.149214539	1.103809503	UP
Isoindoline	0.016137066	0.801394689	-0.319415146	DOWN
12-Hydroxydodecanoic acid	0.01371213	0.526329357	-0.925962228	DOWN
3-Hexenedioic acid	0.009479735	7.052039265	2.818040507	UP
Cis-Chrysanthemol	0.008717944	0.651794036	-0.617511944	DOWN
Mevalonic acid	0.016520362	24.15760246	4.594405375	UP
3'-Deoxycapsanthin	0.01231699	0.555777941	-0.84741952	DOWN
Coniferin	0.011318309	3.278208584	1.712907652	UP
4-Chlorobenzophenone	0.015881879	0.363525326	-1.459872219	DOWN
Arachidoyl Ethanolamide	0.021615397	0.639382023	-0.645249913	DOWN
Diethanolamine	0.011249248	0.455562452	-1.134279251	DOWN
Retinol	0.01301567	36.69861397	5.197653671	UP
Prolylarginine	0.014783376	5.630651658	2.493301901	UP
Clupanodonic acid	0.006680274	3.198204751	1.677262304	UP
N-oleoyl leucine	0.00982887	2.734485998	1.451269675	UP
1-Hydroxypyrrolidine-3-carboxylic acid	0.007627059	1.867860139	0.901386433	UP
Linoleoyl Ethanolamide	0.022356107	3.513268667	1.812813907	UP
5(S)-HETE	0.006857896	0.458709276	-1.124348013	DOWN

LPC(16:0)	0.010088253	0.782710579	-0.353449151	DOWN
7-Methylguanosine	0.015182606	3.692625054	1.88464678	UP
N-butyl-N-(1-(cyclohexylamino)-1-oxobutan-2-yl)butyramide	0.007682279	0.421279711	-1.247149659	DOWN
5-Hydroxymethylcytosine	0.009402015	1.423948128	0.509896592	UP
Fumonisin A1	0.022706734	0.310450403	-1.687565288	DOWN
hydroxydeoxyguanosine	0.021088171	4.77954778	2.256874123	UP
4-Oxo-L-proline	0.014008725	3.76957744	1.91440281	UP
2-oxo-1H-quinoline-4-carboxylic acid	0.010529491	2.53955177	1.344573885	UP
GABA	0.014256453	2.237019871	1.161578072	UP
4-[(E)-3-Hydroxy-8,10-dimethyl-2-(methylamino)dodec-6-enyl]phenol	0.0111696	0.557864558	-0.842013198	DOWN
4-(Butylamino)benzoic acid	0.019800773	2.262986264	1.178227828	UP
2-Hexenoylcarnitine	0.019603253	2.916470475	1.544223469	UP
Cordycepin	0.035850143	0.740708067	-0.433023044	DOWN
2-pyridin-3-yl-thiazolidine-4-carboxylic acid	0.021797917	2.990287358	1.58028413	UP
Dimethenamide ESA	0.027561554	4.537772431	2.18198426	UP
Aerugine	0.01665698	14.00703604	3.808079801	UP
15-Deoxy-PGJ2	0.039908504	1.556041911	0.637880919	UP
8-[1-(acetyloxy)-4-oxo-5-[(3E)-2-oxopent-3-en-1-yl]cyclopent-2-en-1-yl]octanoic acid	0.019356874	2.881433291	1.52678662	UP
Trimipramine maleate salt	0.013313578	1.531988187	0.615405173	UP
Sphingosine	0.022111928	0.685500295	-0.544770809	DOWN
Cinnamic acid	0.021821917	0.605639264	-0.723469356	DOWN
7-Dehydrocholesterol	0.016111202	5.688084404	2.507942873	UP
trans-Cinnamoyl beta-D-glucoside	0.015327195	3.894339247	1.961378567	UP
Gentiobiose	0.01284627	0.655520681	-0.609286798	DOWN
Taurochenodeoxycholic acid	0.020584199	0.477174734	-1.067410438	DOWN
Maackiain	0.024397498	0.6520115	-0.617030684	DOWN
Creatinine	0.021113798	2.49302849	1.31789937	UP
Hexadecanamide	0.020925688	1.680269679	0.748692801	UP
Phthalic anhydride	0.018804979	2.1185752	1.083094339	UP
Ehretioside B	0.015660811	4.014236324	2.005125553	UP
Nonactinic Acid	0.025674851	1.888588612	0.917308476	UP
Leucylglutamate	0.018379105	0.562423964	-0.830270028	DOWN

2-Methylpropanoic acid	0.029118485	4.751862621	2.248493128	UP
Capric dimethyl amine oxide	0.037279103	0.689458477	-0.536464428	DOWN
LPE(14:0)	0.044452207	1.744094861	0.80247851	UP
Itaconic acid	0.02953753	2.18089013	1.124917091	UP
Prolylleucine	0.014869664	2.911413457	1.541719735	UP
Tolterodine tartrate	0.038972535	12.76302649	3.67389857	UP
Tetra(methoxymethyl)melamine	0.025415424	3.291037049	1.718542268	UP
Guaiacol	0.017415098	1.567921606	0.648853428	UP
Pyrimido[1,2-A]Purin-10(1H)-One	0.033308789	0.64110836	-0.641359874	DOWN
Heptadecanoyl Ethanolamide	0.024953716	2.515912422	1.331081703	UP
DG(18:1/18:1)	0.024202129	0.038596065	-4.695402422	DOWN
(3Z,9Z)-Hexadecadienoylcarnitine	0.022907384	0.347091798	-1.526610823	DOWN
LPC(18:3)	0.015236609	0.565118481	-0.823374724	DOWN
13,14-Dihydro-15-keto-PGE2	0.041255469	2.33042793	1.220594897	UP
Zaluzanin D	0.033521914	0.701947519	-0.510564923	DOWN
Stearic acid amide	0.037440552	1.906001235	0.930549054	UP
4-methyl-6-(1,2,3,4-tetrahydroquinolylsulfonyl)hydroquinolin-2-one	0.032569722	0.556404756	-0.845793344	DOWN
6-amino-4-(4-hydroxyphenyl)-3-propyl-4H-pyrano[3,2-d]pyrazole-5-carbonitrile	0.023422177	0.689139985	-0.537131028	DOWN
Propionylcarnitine	0.025489265	3.436983637	1.781142983	UP
N2,N2-Dimethylguanosine	0.016535331	1.997038616	0.99786223	UP
1-Methyladenosine	0.027111034	2.025177293	1.018048214	UP
1-Aminomethylphosphonic acid	0.021516954	84.56799992	6.402039953	UP
2-Hydroxy-4-imino-2,5-cyclohexadienone	0.026940996	2.624045946	1.391792981	UP
Cholylvaline	0.021372008	0.383401989	-1.383070275	DOWN
Crotonosine	0.037414062	0.632016955	-0.661964832	DOWN
9-Cyclopentyladenine monomethanesulfonate	0.016997156	2.486069665	1.313866724	UP
(10E,12Z)-Octadecadienoic acid	0.03996333	1.55055281	0.632782663	UP
Kyotorphin	0.041701293	0.604249731	-0.72678317	DOWN
N-(2-Hydroxy-2-Phenylethyl)-2-((4-(4-Methoxyphenyl)-2-Oxo-2H-Chromen-7-Yl)Oxy)Acetamide	0.025751381	20.29751652	4.343231314	UP
Arabinose	0.027192582	2.443595063	1.289005231	UP
cis-Aconitic anhydride	0.013931448	2.1804988	1.124658196	UP
Glutamyltyrosine	0.027478298	1.967635369	0.976462893	UP

2-(2-amino-3-carbamoylpropanamido)-3-phenylpropanoic acid	0.019721942	3.565082461	1.833935447	UP
1,2-dihydroxyheptadec-16-yn-4-yl acetate	0.026223911	7.258169362	2.859605721	UP
2-Methylbenzaldehyde	0.033621711	0.626965218	-0.673542685	DOWN
Betaine	0.023967111	0.608904168	-0.715712907	DOWN
Tricholide A	0.038044956	0.559688062	-0.837305119	DOWN
[3-Methyl-1-[3-methyl-1-oxo-1-(2,3,4,5,6-pentahydroxyhexoxy)pentan-2-yl]oxy-1-oxopentan-2-yl] 2-hydroxy-3-methylpentanoate	0.027163551	0.516937922	-0.951937053	DOWN
Glutaric acid	0.029650073	2.541235911	1.345530312	UP
Allose	0.031280338	2.059879879	1.04256021	UP
LPE(18:1)	0.043784779	1.352249756	0.435361638	UP
Kobusone	0.023061579	0.660778868	-0.597760546	DOWN
4-Pyridoxate	0.043718425	1.855169371	0.891550907	UP
Pellitorine	0.018125506	0.583085541	-0.778220547	DOWN
Pinthunamide	0.038577665	1.94362074	0.958746732	UP
Leu-leu-ome	0.036624767	1.933617314	0.951302296	UP
Butyrolactone III	0.029818139	4.26824527	2.093643081	UP
Tiglylcarnitine	0.032438712	3.526237305	1.818129567	UP
2-amino-3-hydroxynonanoic acid	0.033021997	3.553930876	1.829415621	UP
Biotin	0.026913751	2.069574489	1.049334175	UP
Isoleucylarginine	0.042881079	10.23720024	3.355749303	UP
LPC(22:6)	0.017502366	0.601624537	-0.733064686	DOWN
2,2-Dimethylsuccinic acid	0.038360399	3.417275313	1.772846483	UP
N-Octyl-2-pyrrolidone	0.046703137	0.358841322	-1.478582063	DOWN
2-Acetolactate	0.04367808	2.793166355	1.4819015	UP
Glycerophosphocholine	0.04539943	1.422157876	0.508081629	UP
Tropic acid	0.038851668	0.63219193	-0.661565475	DOWN
4-Methylheptanoylcarnitine	0.046868977	0.355418043	-1.492411172	DOWN
Methylpyrrolidone	0.042273357	1.274779752	0.350248009	UP
Dodecyl sulfate	0.045891349	0.704256951	-0.505826197	DOWN
Acetaminophen sulfate ester	0.043247163	2.020748153	1.014889529	UP
Kynurenic acid	0.036400406	2.782793414	1.476533811	UP
Inosine	0.029518648	1.963579226	0.973485809	UP
2-Methyl-4'-(methylthio)-2-morpholinopropiophenone	0.019423057	2.477770049	1.309042304	UP

Andrachcinidine	0.029117691	2.464775424	1.301456203	UP
PS(18:1/18:0)	0.046787156	0.391376858	-1.353369641	DOWN
2-(1H-indol-3-yl)-2-oxo-N-[(1R)-1-phenylethyl]acetamide	0.028054659	3.789466472	1.921994742	UP
N-ethyl-2-(6-oxo-9-phenylhydropurin-8-ylthio)-N-phenylacetamide	0.039032416	2.427859324	1.279684831	UP
Heptadecanoyl carnitine	0.034927495	0.571606144	-0.806906671	DOWN
Indole-3-ethanol	0.027596	1.361209996	0.44488965	UP
Mevalonolactone	0.037682772	1.40447912	0.490035176	UP
Acetylcarnitine	0.036492121	3.155287357	1.6577714	UP
[(2E,6E)-1-Oxo-8-hydroxy-2,6-dimethylocta-2,6-dien-1-yl]beta-D-glucopyranoside	0.033192278	4.098621366	2.035138719	UP
N-Methylcalystegine C1	0.025386662	0.676286593	-0.564293341	DOWN
Butyrylcarnitine	0.038780811	2.842862318	1.50734423	UP
N-[3-(trifluoromethyl)phenyl]quinazolin-4-amine	0.044979673	3.099078931	1.6318395	UP
1,3-Dimethyluric acid	0.046464306	2.349222276	1.232183223	UP
Jaeschkeanadiol	0.039893945	9.302103356	3.21755697	UP
cis-Dihydrocarvone	0.040761669	2.426709405	1.279001358	UP
Pirbuterol	0.049094798	2.772702251	1.4712927	UP
1-(2H-benzo[3,4-d]1,3-dioxolan-5-yl)-3-[(4-chlorophenyl)amino]-3-(4-ethylpheny l)propan-1-one	0.03128422	2.127607401	1.089231961	UP
(2E)-N-[4-(acetylamino)phenyl]-3-phenylprop-2-enamide	0.043185424	2.803719912	1.487342233	UP
Proline	0.048680353	0.713686746	-0.486637115	DOWN
Adenosine	0.041168073	3.431442754	1.778815287	UP
3-Hydroxy-2-[[3-(3-hydroxy-6-methyloctanoyl)oxy-8-methylnonanoyl]amino]propanoic acid	0.032499455	0.633626232	-0.658296031	DOWN
Capryloyl glycine	0.04180708	1.734900022	0.794852526	UP
Rhodiocyanoside A	0.04137253	1.954792702	0.967015623	UP
cis-5-Tetradecenoylcarnitine	0.035115359	0.376034769	-1.41106203	DOWN
N-Methylglutamic acid	0.042247032	1.929928682	0.948547536	UP
-Icosa-8,11,14,17-tetraenoylcarnitine	0.03258127	0.322297497	-1.633535109	DOWN
N-Acetylmethionine	0.042398553	2.295299305	1.198682292	UP
1-Aminocyclopentane-1,3-Dicarboxylic Acid	0.043117468	3.874235312	1.953911583	UP
N-(2-octyloxyethyl)(3,4,5-trimethoxyphenyl)carboxamide	0.035040261	2.780079742	1.475126265	UP
2-Naphthalenemethanol, 7-(acetyloxy)decahydro-alpha,alpha,4a-trimethyl-8-methylene-	0.031687737	0.592002899	-0.756323855	DOWN
1-(2-hydroxy-3-methyl)-butyl-hydrocotarnine	0.040281798	0.560398173	-0.835475844	DOWN
Caffeine	0.035005664	2.567672267	1.360461071	UP

Glycylleucine	0.04673101	2.165406408	1.114637818	UP
Isocitric acid	0.038751946	2.193074195	1.132954621	UP
Leucyl-Lysine	0.046673896	0.308435281	-1.696960295	DOWN
2-Hydroxy-4-methoxy-6-pentyl-benzoesaeure	0.040278391	0.583003071	-0.778424613	DOWN
2,2',4,4',6,6'-hexamethoxy-1,1'-biphenyl	0.029440733	0.531596633	-0.911596127	DOWN
Citraconic anhydride	0.044896996	1.894932661	0.922146581	UP
Butyl 2-amino-4-methyl-pentanoate	0.032028176	0.534506967	-0.903719343	DOWN
Cyclo(Pro-Thr)	0.036823502	2.488198677	1.315101686	UP
Linolenic acid	0.038623083	1.753491963	0.810230818	UP
Diacetyl	0.030846034	1.690824082	0.757726565	UP
N-Oleoyldopamine	0.041173525	22.51509833	4.492820873	UP
1,4-Cyclohexanedicarboxylic acid	0.035561058	0.600623894	-0.735466226	DOWN
Aminoadipic acid	0.046341543	0.600449851	-0.735884336	DOWN
Antheraxanthin	0.038935451	4.733760589	2.242986743	UP
Pivaloylcarnitine	0.046072732	3.444944118	1.78448058	UP
LPC(20:5)	0.030957959	0.623902683	-0.680607081	DOWN
2-(N-butylacetamido)-N-cyclohexylbutanamide	0.037424129	0.597466048	-0.743071362	DOWN
Pipecolic acid	0.035243793	1.577015451	0.657196796	UP
Ronidazole	0.040370587	0.522333311	-0.936957383	DOWN
