# Supplementary material for: Liquid chromatograph-mass spectrometry metabolomics uncovers potential biomarkers of semen cryo-injury in goats
Source: Anim Biosci. 2024 Oct 28;38(4):629–40. doi: 10.5713/ab.24.0435 (PMC11917422; doi:10.5713/ab.24.0435)
Supplement: Supplementary file 3 [file ab-24-0435-Supplementary-Table-3.pdf]

Supplementary Table S3. Significant KEGG metabolic pathways based on p<0.05.

Description	MetaboRatio	BgRatio	pvalue
Protein digestion and absorption	11/77	47/4432	2.20784E-10
Central carbon metabolism in cancer	8/77	37/4432	1.47689E-07
Regulation of lipolysis in adipocytes	5/77	14/4432	2.46078E-06
ABC transporters	12/77	138/4432	3.33996E-06
Biosynthesis of amino acids	11/77	128/4432	1.00161E-05
Choline metabolism in cancer	4/77	11/4432	2.53504E-05
Aminoacyl-tRNA biosynthesis	7/77	52/4432	2.59162E-05
Mineral absorption	5/77	29/4432	0.000119033
African trypanosomiasis	3/77	8/4432	0.000265279
Vitamin digestion and absorption	5/77	39/4432	0.000503923
Cholesterol metabolism	3/77	10/4432	0.000554349
cAMP signaling pathway	4/77	25/4432	0.000807716
Rheumatoid arthritis	2/77	3/4432	0.000883882
Oxytocin signaling pathway	3/77	12/4432	0.000991123
Amoebiasis	3/77	13/4432	0.001272413
Biosynthesis of unsaturated fatty acids	6/77	74/4432	0.001639094
Sphingolipid signaling pathway	3/77	15/4432	0.001974236
2-Oxocarboxylic acid metabolism	8/77	134/4432	0.002057084
Neuroactive ligand-receptor interaction	5/77	53/4432	0.002078569
Vascular smooth muscle contraction	3/77	16/4432	0.002399631
Glycerophospholipid metabolism	5/77	56/4432	0.002657049
Phenylalanine, tyrosine and tryptophan biosynthesis	4/77	35/4432	0.002931525
Inflammatory mediator regulation of TRP channels	4/77	35/4432	0.002931525
Retrograde endocannabinoid signaling	3/77	19/4432	0.003999534
GnRH signaling pathway	2/77	6/4432	0.004271822
Melanogenesis	2/77	6/4432	0.004271822
Leishmaniasis	2/77	6/4432	0.004271822
Human cytomegalovirus infection	2/77	6/4432	0.004271822
Serotonergic synapse	4/77	42/4432	0.005717912
Bile secretion	6/77	97/4432	0.006380691

D-Amino acid metabolism	5/77	69/4432	0.006562821
Fc gamma R-mediated phagocytosis	2/77	8/4432	0.007796096
Estrogen signaling pathway	2/77	8/4432	0.007796096
Morphine addiction	2/77	8/4432	0.007796096
Glycine, serine and threonine metabolism	4/77	48/4432	0.009191234
Parkinson disease	3/77	26/4432	0.009836304
Long-term depression	2/77	9/4432	0.009911281
GnRH secretion	2/77	9/4432	0.009911281
Arachidonic acid metabolism	5/77	79/4432	0.011509269
Alanine, aspartate and glutamate metabolism	3/77	28/4432	0.012090129
Necroptosis	2/77	10/4432	0.012250514
Alcoholism	2/77	10/4432	0.012250514
Ferroptosis	3/77	29/4432	0.013319132
C-type lectin receptor signaling pathway	2/77	11/4432	0.014805578
Fc epsilon RI signaling pathway	2/77	11/4432	0.014805578
Lysine degradation	4/77	56/4432	0.015634421
Cholinergic synapse	2/77	12/4432	0.017568469
Dopaminergic synapse	2/77	12/4432	0.017568469
Fat digestion and absorption	2/77	13/4432	0.020531388
Platelet activation	2/77	14/4432	0.02368674
Arginine and proline metabolism	4/77	69/4432	0.031100529
Renin secretion	2/77	17/4432	0.034234364
EGFR tyrosine kinase inhibitor resistance	1/77	2/4432	0.034449302
Insulin resistance	2/77	19/4432	0.042097702
Citrate cycle (TCA cycle)	2/77	20/4432	0.046258889
Primary bile acid biosynthesis	3/77	47/4432	0.047370547
Metabolic pathways	60/77	3048/4432	0.049086816
