# Supplementary material for: Liquid chromatograph-mass spectrometry metabolomics uncovers potential biomarkers of semen cryo-injury in goats
Source: Anim Biosci. 2024 Oct 28;38(4):629–40. doi: 10.5713/ab.24.0435 (PMC11917422; doi:10.5713/ab.24.0435)
Supplement: Supplementary file 4 [file ab-24-0435-Supplementary-Table-4.pdf]

Supplementary Table S4. Enriched KEGG metabolic pathways and their classifications.

Description	MetaboRatio	BgRatio	pvalue	SecondClass	TopClass
Protein digestion and absorption	11/77	47/4432	2.20784E-10	Digestive system	Organismal Systems
Central carbon metabolism in cancer	8/77	37/4432	1.47689E-07	Cancer: overview	Human Diseases
Regulation of lipolysis in adipocytes	5/77	14/4432	2.46078E-06	Endocrine system	Organismal Systems
ABC transporters	12/77	138/4432	3.33996E-06	Membrane transport	Environmental Information Processing
Biosynthesis of amino acids	11/77	128/4432	1.00161E-05	Global and overview maps	Metabolism
Choline metabolism in cancer	4/77	11/4432	2.53504E-05	Cancer: overview	Human Diseases
Aminoacyl-tRNA biosynthesis	7/77	52/4432	2.59162E-05	Translation	Genetic Information Processing
Mineral absorption	5/77	29/4432	0.000119033	Digestive system	Organismal Systems
African trypanosomiasis	3/77	8/4432	0.000265279	Infectious disease: parasitic	Human Diseases
Vitamin digestion and absorption	5/77	39/4432	0.000503923	Digestive system	Organismal Systems
Cholesterol metabolism	3/77	10/4432	0.000554349	Digestive system	Organismal Systems
cAMP signaling pathway	4/77	25/4432	0.000807716	Signal transduction	Environmental Information Processing
Rheumatoid arthritis	2/77	3/4432	0.000883882	Immune disease	Human Diseases
Oxytocin signaling pathway	3/77	12/4432	0.000991123	Endocrine system	Organismal Systems
Amoebiasis	3/77	13/4432	0.001272413	Infectious disease: parasitic	Human Diseases
Biosynthesis of unsaturated fatty acids	6/77	74/4432	0.001639094	Lipid metabolism	Metabolism
Sphingolipid signaling pathway	3/77	15/4432	0.001974236	Signal transduction	Environmental Information Processing
2-Oxocarboxylic acid metabolism	8/77	134/4432	0.002057084	Global and overview maps	Metabolism
Neuroactive ligand-receptor interaction	5/77	53/4432	0.002078569	Signaling molecules and interaction	Environmental Information Processing
Vascular smooth muscle contraction	3/77	16/4432	0.002399631	Circulatory system	Organismal Systems
Glycerophospholipid metabolism	5/77	56/4432	0.002657049	Lipid metabolism	Metabolism
Phenylalanine, tyrosine and tryptophan biosynthesis	4/77	35/4432	0.002931525	Amino acid metabolism	Metabolism
Inflammatory mediator regulation of TRP channels	4/77	35/4432	0.002931525	Sensory system	Organismal Systems
Retrograde endocannabinoid signaling	3/77	19/4432	0.003999534	Nervous system	Organismal Systems
GnRH signaling pathway	2/77	6/4432	0.004271822	Endocrine system	Organismal Systems
Melanogenesis	2/77	6/4432	0.004271822	Endocrine system	Organismal Systems
Leishmaniasis	2/77	6/4432	0.004271822	Infectious disease: parasitic	Human Diseases

Human cytomegalovirus infection	2/77	6/4432	0.004271822	Infectious disease: viral	Human Diseases
Serotonergic synapse	4/77	42/4432	0.005717912	Nervous system	Organismal Systems
Bile secretion	6/77	97/4432	0.006380691	Digestive system	Organismal Systems
D-Amino acid metabolism	5/77	69/4432	0.006562821	Metabolism of other amino acids	Metabolism
Fc gamma R-mediated phagocytosis	2/77	8/4432	0.007796096	Immune system	Organismal Systems
Estrogen signaling pathway	2/77	8/4432	0.007796096	Endocrine system	Organismal Systems
Morphine addiction	2/77	8/4432	0.007796096	Substance dependence	Human Diseases
Glycine, serine and threonine metabolism	4/77	48/4432	0.009191234	Amino acid metabolism	Metabolism
Parkinson disease	3/77	26/4432	0.009836304	Neurodegenerative disease	Human Diseases
Long-term depression	2/77	9/4432	0.009911281	Nervous system	Organismal Systems
GnRH secretion	2/77	9/4432	0.009911281	Endocrine system	Organismal Systems
Arachidonic acid metabolism	5/77	79/4432	0.011509269	Lipid metabolism	Metabolism
Alanine, aspartate and glutamate metabolism	3/77	28/4432	0.012090129	Amino acid metabolism	Metabolism
Necroptosis	2/77	10/4432	0.012250514	Cell growth and death	Cellular Processes
Alcoholism	2/77	10/4432	0.012250514	Substance dependence	Human Diseases
Ferroptosis	3/77	29/4432	0.013319132	Cell growth and death	Cellular Processes
C-type lectin receptor signaling pathway	2/77	11/4432	0.014805578	Immune system	Organismal Systems
Fc epsilon RI signaling pathway	2/77	11/4432	0.014805578	Immune system	Organismal Systems
Lysine degradation	4/77	56/4432	0.015634421	Amino acid metabolism	Metabolism
Cholinergic synapse	2/77	12/4432	0.017568469	Nervous system	Organismal Systems
Dopaminergic synapse	2/77	12/4432	0.017568469	Nervous system	Organismal Systems
Fat digestion and absorption	2/77	13/4432	0.020531388	Digestive system	Organismal Systems
Platelet activation	2/77	14/4432	0.02368674	Immune system	Organismal Systems
Arginine and proline metabolism	4/77	69/4432	0.031100529	Amino acid metabolism	Metabolism
Renin secretion	2/77	17/4432	0.034234364	Endocrine system	Organismal Systems
EGFR tyrosine kinase inhibitor resistance	1/77	2/4432	0.034449302	Drug resistance: antineoplastic	Human Diseases
Insulin resistance	2/77	19/4432	0.042097702	Endocrine and metabolic disease	Human Diseases
Citrate cycle (TCA cycle)	2/77	20/4432	0.046258889	Carbohydrate metabolism	Metabolism
Primary bile acid biosynthesis	3/77	47/4432	0.047370547	Lipid metabolism	Metabolism
Metabolic pathways	60/77	3048/4432	0.049086816	Global and overview maps	Metabolism
NF-kappa B signaling pathway	1/77	3/4432	0.051232011	Signal transduction	Environmental Information Processing
Th1 and Th2 cell differentiation	1/77	3/4432	0.051232011	Immune system	Organismal Systems

Human papillomavirus infection	1/77	3/4432	0.051232011	Infectious disease: viral	Human Diseases
Aldosterone synthesis and secretion	2/77	22/4432	0.05500878	Endocrine system	Organismal Systems
Tryptophan metabolism	4/77	83/4432	0.055224149	Amino acid metabolism	Metabolism
Thermogenesis	2/77	23/4432	0.05958538	Environmental adaptation	Organismal Systems
Taurine and hypotaurine metabolism	2/77	24/4432	0.064288617	Metabolism of other amino acids	Metabolism
ErbB signaling pathway	1/77	4/4432	0.067726736	Signal transduction	Environmental Information Processing
Apoptosis	1/77	4/4432	0.067726736	Cell growth and death	Cellular Processes
Natural killer cell mediated cytotoxicity	1/77	4/4432	0.067726736	Immune system	Organismal Systems
Th17 cell differentiation	1/77	4/4432	0.067726736	Immune system	Organismal Systems
T cell receptor signaling pathway	1/77	4/4432	0.067726736	Immune system	Organismal Systems
B cell receptor signaling pathway	1/77	4/4432	0.067726736	Immune system	Organismal Systems
Growth hormone synthesis, secretion and action	1/77	4/4432	0.067726736	Endocrine system	Organismal Systems
Glioma	1/77	4/4432	0.067726736	Cancer: specific types	Human Diseases
PD-L1 expression and PD-1 checkpoint pathway in cancer	1/77	4/4432	0.067726736	Cancer: overview	Human Diseases
Ether lipid metabolism	2/77	25/4432	0.069112841	Lipid metabolism	Metabolism
Glucagon signaling pathway	2/77	26/4432	0.074052552	Endocrine system	Organismal Systems
Steroid biosynthesis	3/77	57/4432	0.075697128	Lipid metabolism	Metabolism
Sphingolipid metabolism	2/77	27/4432	0.079102403	Lipid metabolism	Metabolism
MAPK signaling pathway	1/77	5/4432	0.083938353	Signal transduction	Environmental Information Processing
Rap1 signaling pathway	1/77	5/4432	0.083938353	Signal transduction	Environmental Information Processing
Chemokine signaling pathway	1/77	5/4432	0.083938353	Immune system	Organismal Systems
Neurotrophin signaling pathway	1/77	5/4432	0.083938353	Nervous system	Organismal Systems
Kaposi sarcoma-associated herpesvirus infection	1/77	5/4432	0.083938353	Infectious disease: viral	Human Diseases
Human immunodeficiency virus 1 infection	1/77	5/4432	0.083938353	Infectious disease: viral	Human Diseases
Asthma	1/77	5/4432	0.083938353	Immune disease	Human Diseases
Linoleic acid metabolism	2/77	28/4432	0.084257194	Lipid metabolism	Metabolism
Biotin metabolism	2/77	29/4432	0.089511865	Metabolism of cofactors and vitamins	Metabolism
VEGF signaling pathway	1/77	6/4432	0.099871659	Signal transduction	Environmental Information Processing
Relaxin signaling pathway	1/77	6/4432	0.099871659	Endocrine system	Organismal Systems
Non-small cell lung cancer	1/77	6/4432	0.099871659	Cancer: specific types	Human Diseases

Pathways in cancer	2/77	31/4432	0.100301315	Cancer: overview	Human Diseases
Taste transduction	2/77	32/4432	0.105826665	Sensory system	Organismal Systems
Ras signaling pathway	1/77	7/4432	0.115531371	Signal transduction	Environmental Information Processing
Long-term potentiation	1/77	7/4432	0.115531371	Nervous system	Organismal Systems
Adipocytokine signaling pathway	1/77	7/4432	0.115531371	Endocrine system	Organismal Systems
Spinocerebellar ataxia	1/77	7/4432	0.115531371	Neurodegenerative disease	Human Diseases
Cocaine addiction	1/77	7/4432	0.115531371	Substance dependence	Human Diseases
Nicotine addiction	1/77	7/4432	0.115531371	Substance dependence	Human Diseases
Glutamatergic synapse	1/77	8/4432	0.130922124	Nervous system	Organismal Systems
Endocrine and other factor-regulated calcium reabsorption	1/77	8/4432	0.130922124	Excretory system	Organismal Systems
Apelin signaling pathway	1/77	9/4432	0.14604848	Signal transduction	Environmental Information Processing
Circadian entrainment	1/77	9/4432	0.14604848	Environmental adaptation	Organismal Systems
GABAergic synapse	1/77	9/4432	0.14604848	Nervous system	Organismal Systems
AGE-RAGE signaling pathway in diabetic complications	1/77	9/4432	0.14604848	Endocrine and metabolic disease	Human Diseases
Amphetamine addiction	1/77	9/4432	0.14604848	Substance dependence	Human Diseases
cGMP-PKG signaling pathway	1/77	10/4432	0.16091492	Signal transduction	Environmental Information Processing
Adrenergic signaling in cardiomyocytes	1/77	10/4432	0.16091492	Circulatory system	Organismal Systems
Parathyroid hormone synthesis, secretion and action	1/77	10/4432	0.16091492	Endocrine system	Organismal Systems
Coronavirus disease - COVID-19	1/77	10/4432	0.16091492	Infectious disease: viral	Human Diseases
Calcium signaling pathway	1/77	11/4432	0.175525855	Signal transduction	Environmental Information Processing
Phospholipase D signaling pathway	1/77	11/4432	0.175525855	Signal transduction	Environmental Information Processing
Sulfur relay system	1/77	11/4432	0.175525855	Folding, sorting and degradation	Genetic Information Processing
Gap junction	1/77	11/4432	0.175525855	Cellular community - eukaryotes	Cellular Processes
Prolactin signaling pathway	1/77	11/4432	0.175525855	Endocrine system	Organismal Systems
Thyroid hormone signaling pathway	1/77	11/4432	0.175525855	Endocrine system	Organismal Systems
Synaptic vesicle cycle	1/77	12/4432	0.189885617	Nervous system	Organismal Systems
Insulin secretion	1/77	12/4432	0.189885617	Endocrine system	Organismal Systems
Butanoate metabolism	2/77	47/4432	0.196185027	Carbohydrate metabolism	Metabolism

Neutrophil extracellular trap formation	1/77	13/4432	0.203998469	Immune system	Organismal Systems
Biosynthesis of cofactors	8/77	328/4432	0.207397601	Global and overview maps	Metabolism
Phenylalanine metabolism	2/77	49/4432	0.208928285	Amino acid metabolism	Metabolism
Fatty acid degradation	2/77	50/4432	0.215333496	Lipid metabolism	Metabolism
Gastric acid secretion	1/77	14/4432	0.217868602	Digestive system	Organismal Systems
Hepatocellular carcinoma	1/77	14/4432	0.217868602	Cancer: specific types	Human Diseases
HIF-1 signaling pathway	1/77	15/4432	0.231500136	Signal transduction	Environmental Information Processing
Pancreatic secretion	1/77	15/4432	0.231500136	Digestive system	Organismal Systems
Salivary secretion	1/77	17/4432	0.258063545	Digestive system	Organismal Systems
Fatty acid biosynthesis	2/77	58/4432	0.267049833	Lipid metabolism	Metabolism
Nucleotide metabolism	2/77	58/4432	0.267049833	Global and overview maps	Metabolism
Glyoxylate and dicarboxylate metabolism	2/77	64/4432	0.305931336	Carbohydrate metabolism	Metabolism
Thyroid hormone synthesis	1/77	21/4432	0.30850095	Endocrine system	Organismal Systems
Carbon metabolism	3/77	114/4432	0.317871622	Global and overview maps	Metabolism
Caffeine metabolism	1/77	22/4432	0.320572006	Biosynthesis of other secondary metabolites	Metabolism
Valine, leucine and isoleucine biosynthesis	1/77	23/4432	0.332435034	Amino acid metabolism	Metabolism
Ovarian steroidogenesis	1/77	24/4432	0.344093574	Endocrine system	Organismal Systems
Ubiquinone and other terpenoid-quinone biosynthesis	2/77	71/4432	0.350816034	Metabolism of cofactors and vitamins	Metabolism
Retinol metabolism	1/77	25/4432	0.355551105	Metabolism of cofactors and vitamins	Metabolism
Selenocompound metabolism	1/77	27/4432	0.377876764	Metabolism of other amino acids	Metabolism
Carbohydrate digestion and absorption	1/77	27/4432	0.377876764	Digestive system	Organismal Systems
Tyrosine metabolism	2/77	78/4432	0.394725736	Amino acid metabolism	Metabolism
Vitamin B6 metabolism	1/77	29/4432	0.399438695	Metabolism of cofactors and vitamins	Metabolism
Thiamine metabolism	1/77	31/4432	0.420262692	Metabolism of cofactors and vitamins	Metabolism
beta-Alanine metabolism	1/77	32/4432	0.430405789	Metabolism of other amino acids	Metabolism
Pathways of neurodegeneration - multiple diseases	1/77	32/4432	0.430405789	Neurodegenerative disease	Human Diseases
Sulfur metabolism	1/77	33/4432	0.440373688	Energy metabolism	Metabolism
Glutathione metabolism	1/77	38/4432	0.487688001	Metabolism of other amino acids	Metabolism
Glycerolipid metabolism	1/77	38/4432	0.487688001	Lipid metabolism	Metabolism
Diabetic cardiomyopathy	1/77	39/4432	0.496665704	Cardiovascular disease	Human Diseases
Valine, leucine and isoleucine degradation	1/77	42/4432	0.522677476	Amino acid metabolism	Metabolism

Purine metabolism	2/77	101/4432	0.528173581	Nucleotide metabolism	Metabolism
alpha-Linolenic acid metabolism	1/77	44/4432	0.539276847	Lipid metabolism	Metabolism
Terpenoid backbone biosynthesis	1/77	46/4432	0.555306197	Metabolism of terpenoids and polyketides	Metabolism
Fructose and mannose metabolism	1/77	55/4432	0.620887507	Carbohydrate metabolism	Metabolism
Nicotinate and nicotinamide metabolism	1/77	55/4432	0.620887507	Metabolism of cofactors and vitamins	Metabolism
Ascorbate and aldarate metabolism	1/77	57/4432	0.63411034	Carbohydrate metabolism	Metabolism
Chemical carcinogenesis - reactive oxygen species	1/77	57/4432	0.63411034	Cancer: overview	Human Diseases
Pentose and glucuronate interconversions	1/77	58/4432	0.640549998	Carbohydrate metabolism	Metabolism
Cysteine and methionine metabolism	1/77	67/4432	0.693695393	Amino acid metabolism	Metabolism
Amino sugar and nucleotide sugar metabolism	1/77	118/4432	0.877070916	Carbohydrate metabolism	Metabolism
Fatty acid metabolism	1/77	122/4432	0.885618192	Global and overview maps	Metabolism
Porphyrin metabolism	1/77	148/4432	0.928516662	Metabolism of cofactors and vitamins	Metabolism
Biosynthesis of nucleotide sugars	1/77	200/4432	0.972322747	Global and overview maps	Metabolism
