# Supplementary material for: Liquid chromatograph-mass spectrometry metabolomics uncovers potential biomarkers of semen cryo-injury in goats
Source: Anim Biosci. 2024 Oct 28;38(4):629–40. doi: 10.5713/ab.24.0435 (PMC11917422; doi:10.5713/ab.24.0435)
Supplement: Supplementary file 5 [file ab-24-0435-Supplementary-Table-5.pdf]

Supplementary Table S5. Significantly enriched metabolic pathways from MSEA ($p<0.05$).

Pathway	Total Cmpd	Hits	Statistic Q	Raw p	FDR	Metabolites
Phosphatidylcholine Biosynthesis	14	2	44.288	0.00014253	0.006499	Phosphorylcholine, Choline
Phospholipid Biosynthesis	29	4	43.586	0.00014941	0.006499	Phosphorylcholine, Choline, Glycerylphosphorylethanolamine, FAD
Vitamin K Metabolism	13	2	38.634	0.00031762	0.009211	FAD, Vitamin K1
Thyroid hormone synthesis	13	2	43.983	0.0014353	0.028577	L-Tyrosine, FAD
Phosphatidylethanolamine Biosynthesis	12	2	43.047	0.0016423	0.028577	Choline, Serine
Phenylalanine and Tyrosine Metabolism	27	4	38.589	0.0025619	0.037147	Phenylalanine, Tyrosine, DOPA, Dopamine
Methionine Metabolism	42	7	29.801	0.0043944	0.047821	Betaine, Adenosine, monophosphate, Serine, L-Homoserine, FAD
Betaine Metabolism	21	4	29.852	0.0043973	0.047821	Betaine, Adenosine, Choline, FAD
Catecholamine Biosynthesis	20	3	33.81	0.0056884	0.054987	Dihydrobiopterin, L-Tyrosine, DOPA
Tyrosine Metabolism	70	7	27.704	0.010907	0.09489	Tyrosine, L-DOPA, Dopamine, Noradrenaline, Adrenaline
Bile Acid Biosynthesis	65	11	17.019	0.013791	0.10384	Taurocholic acid; Cholesterol; Glycocholic acid, Palmitic acid, Lithocholic acid glycine conjugate, Lithocholyltaurine, Lithocholic acid, Taurodeoxycholic acid, FAD, 5b-Cyprinol sulfate
Glutamate Metabolism	48	7	28.787	0.014323	0.10384	Biotin, Adenosine monophosphate, gamma-Aminobutyric acid, Glutamic acid, L-Alanine, L-Aspartic acid, Succinic acid, Glutamine, FAD
Sulfate/Sulfite Metabolism	22	2	19.717	0.034051	0.20163	Phenol, Phenol sulphate
Caffeine Metabolism	23	3	22.251	0.035106	0.20163	FAD, Caffeine, 1,3,7-Trimethyluric acid
Steroidogenesis	43	2	16.711	0.036418	0.20163	Cholesterol, Tetrahydrocortisol
Warburg Effect	57	7	21.558	0.039071	0.20163	Biotin, Citric acid, Fumaric acid, Glutamic acid, Malic acid, Succinic acid, Glutamine, FAD
Citric Acid Cycle	32	8	21.474	0.039398	0.20163	Biotin, cis-Aconitic acid, Citric acid, Fumaric acid, Malic acid, Isocitric acid, Succinic acid, FAD
