# Supplementary material for: Liquid chromatograph-mass spectrometry metabolomics uncovers potential biomarkers of semen cryo-injury in goats
Source: Anim Biosci. 2024 Oct 28;38(4):629–40. doi: 10.5713/ab.24.0435 (PMC11917422; doi:10.5713/ab.24.0435)
Supplement: Supplementary file 6 [file ab-24-0435-Supplementary-Fig-1.pdf]

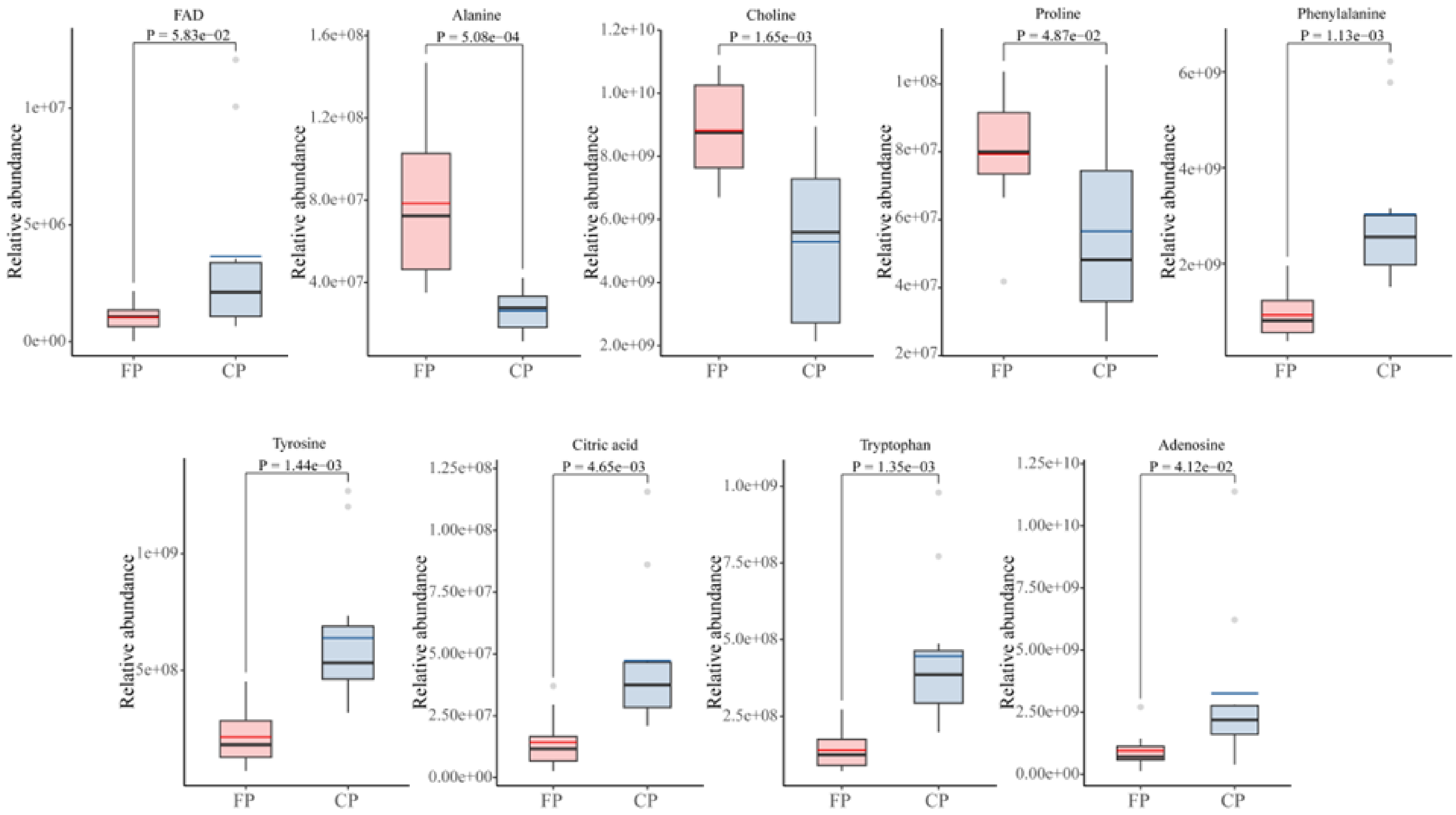

Supplementary Figure S1. Relative abundance of nine selected metabolites in the FP and CP groups.
